# MDFIC2 is a PIEZO channel modulator that can alleviate mechanical allodynia associated with neuropathic pain

**DOI:** 10.1073/pnas.2512426122

**Published:** 2025-11-07

**Authors:** Abdella M. Habib, Shengnan Li, Chenjing Zhang, Meijun Ji, Nancy Osorio, Virginie Penalba, Jesus M. Torres, Samuel J. Gossage, Mehdi A. Rezai, Amy F. Geard, Ahad A. Rahim, Ahmed M. M. Mahmoud, Sonia Santana-Varela, Jun Zhou, Jing Zhao, John N. Wood, Andrei L. Okorokov, Xuelong Zhou, James J. Cox, Bertrand Coste

**Affiliations:** ^a^Department of Basic Medical Sciences, College of Medicine, Qatar University Health, Qatar University, Doha PO Box 2713, Qatar; ^b^Wolfson Institute for Biomedical Research, Division of Medicine, University College London, London WC1E 6BT, United Kingdom; ^c^Center for General Practice Medicine, Department of Gastroenterology, Zhejiang Provincial People’s Hospital (Affiliated People’s Hospital), Hangzhou Medical College, Hangzhou 310014, China; ^d^Aix Marseille University, INSERM 1263, Institut National de Recherche pour l’Agriculture, l’Alimentation et l’Environnement 1260, Centre de recherche en CardioVasculaire et Nutrition, Marseille 13005, France; ^e^CNRS EMR7005, Marseille 13005, France; ^f^Department of Biochemistry, Molecular Biology and Immunology, Faculty of Medicine, University of Granada, Granada 18016, Spain; ^g^Department of Pharmacology, University College London School of Pharmacy, University College London, London WC1N 1AX, United Kingdom; ^h^Department of Medical Pharmacology, Faculty of Medicine, Assiut University, Assiut 71515, Egypt; ^i^Department of Pain, Renmin Hospital of Wuhan University, Wuhan 430060, China; ^j^Department of Anesthesiology, Woman’s Hospital, Zhejiang University School of Medicine, Hangzhou 310006, China

**Keywords:** mechanical allodynia, neuropathic pain, PIEZO channel, dorsal root ganglia

## Abstract

PIEZO channels are mechanical force sensors involved in various biological processes, including somatosensation. To date, only a few PIEZO-binding partners have been identified, including MyoD-family inhibitor proteins (MDFI and MDFIC). Here, we show that MDFIC2, a third member of the MDFI protein family with an as-yet-unknown function, is expressed in a subset of nociceptive sensory neurons. MDFIC2 modulates both PIEZO1 and PIEZO2 gating properties by slowing their kinetics and shifting mechanical sensitivity to higher forces. Interestingly, *Mdfic2* is downregulated in mouse neuropathic pain models in which mechanical allodynia is a hallmark symptom. We found that intrathecal administration of adeno-associated virus vector encoding *MDFIC2* cDNA reduces mechanical sensitivity and attenuates mechanical allodynia in the spared nerve injury neuropathic pain model. These findings demonstrate a mechanism for regulating mechanosensation and highlight a potential therapeutic route for treating mechanical allodynia.

## Significance

Despite the known importance of the PIEZO2 mechanosensitive channel to innocuous touch, proprioception, mechanical pain, and interoception, direct modulators are still underexplored. Here, we identify MDFIC2, a sensory neuron–enriched modulator of PIEZO channels. Mdfic2 is downregulated in mouse neuropathic pain models while viral delivery of Mdfic2 to sensory neurons provides mechanical pain relief. Our findings provide insights into mechanotransduction regulation and highlight a potential analgesic target for chronic pain.

Cells in the body are subject to various mechanical forces, which are detected through mechanotransduction. This process converts mechanical stimuli into biochemical signals and is crucial for cellular functions, organ development, and homeostasis. Among the multiple molecular players involved in cellular mechanotransduction, PIEZO1 and PIEZO2 ion channels have emerged as crucial mediators. PIEZOs are multimodal mechanosensitive channels that respond to various forces, including shear stress, cellular compression, membrane tension, cell swelling, and ultrasound (1–9). Their significance lies in their exquisite sensitivity, broad expression across diverse cell types and tissues, and ability to elicit distinct biological responses depending on downstream signaling pathways. Among their multiple biological functions (10–13), PIEZO channels present in sensory nerve terminals play a central role in somatosensory mechanosensation. PIEZO2, highly expressed in multiple classes of somatosensory neurons, is essential for innocuous touch sensation and proprioception and is also implicated in mechanical nociception (14–18). In contrast, PIEZO1 is selectively expressed in itch-specific sensory neurons, contributing to mechanically evoked scratching behaviors and alloknesis (19).

Although PIEZOs are functional per se in artificial membranes (8), accumulating evidence illustrates that their biophysical properties, including inactivation kinetics and force sensitivity, are regulated by cytoskeletal components and elements of the extracellular matrix (2, 20–23), and lipids (24–27). Furthermore, single point mutations in human PIEZO channels that slightly alter gating properties can lead to diseases such as xerocytosis and distal arthrogryposis (28, 29), demonstrating how subtle changes in channel activity can impact biological functions.

Recently, the transcriptional regulators MyoD (myoblast determination) family-inhibitor proteins MDFI and MDFIC have been shown to modulate the gating properties of both PIEZO1 and PIEZO2, notably by slowing the inactivation and deactivation of the channels (30). MDFI and MDFIC were initially reported as transcriptional regulators (31, 32), and have since been implicated in various biological processes, including lymphatic vasculature development and tumorigenesis (33, 34). The modulation of PIEZO channels relies on the direct interaction of the conserved C-terminal alpha-helix of MDFI/MDFIC with the pore modules of PIEZOs, as revealed by the structural study of the PIEZO1–MDFIC complex (30). Palmitoylation of cysteine residues in the MDFIC alpha-helix is essential for this modulation, with the palmitate chains potentially interacting with amino acids located at the putative inactivation gate of the PIEZO1 channel, as revealed by dynamic simulations (30). Therefore, MDFI/MDFIC proteins could act as auxiliary subunits of PIEZO channels modulating their biological functions. However, since neither MDFI nor MDFIC is expressed in somatosensory neurons, according to public databases (35–38), a role in somatosensation is not expected a priori.

Here, we show that a third member of the MyoD family-inhibitor proteins, MDFIC2, is expressed in subsets of dorsal root ganglia (DRG) sensory neurons. AlphaFold predictions suggest that, similarly to MDFIC, the C-terminal alpha helix of MDFIC2 interacts with PIEZO1. Functional characterization shows that MDFIC2 modulates PIEZO1 and PIEZO2-mediated currents by slowing their kinetics and altering their mechanical sensitivity. Interestingly, the levels of *Mdfic2* mRNA are downregulated in three distinct neuropathic pain models. Behavioral experiments in mice treated with intrathecal adeno-associated virus (AAV) injections demonstrate that *Mdfic2* shRNA induces a slight increase in mouse mechanical sensitivity, while *MDFIC2* cDNA potently and specifically reduces mechanical sensitivity and mechanical allodynia in a spared nerve injury (SNI) model. Our results show that MDFIC2 is a potent modulator of PIEZO channels, potentially involved in the pathogenesis and progression of neuropathic pain.

## Results

### Mdfic2 Is Coexpressed in Piezo2-Positive DRG and Vagal Sensory Neurons.

**Fig. 1.**
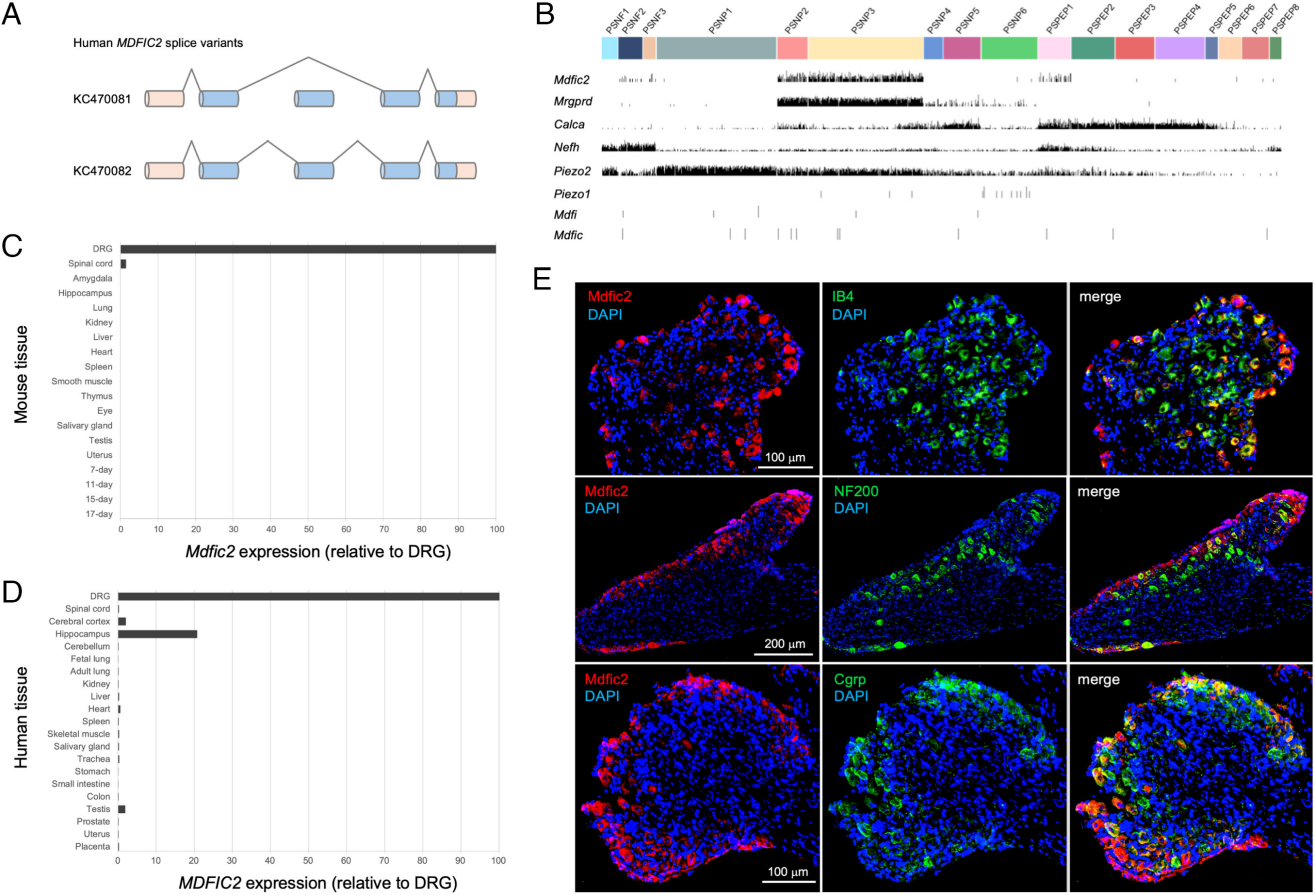
*MDFIC2* splice variants and expression profile. (*A*) Human *MDFIC2* gene is composed of five exons in which exon 3 is alternatively spliced to encode proteins of 189 or 202 amino acids. Blue boxes denote protein-coding sequence. (*B*) Single-cell RNA-seq expression profiles in mouse DRG from https://loom.linnarssonlab.org/. (*C* and *D*) Real-time qPCR assays measuring the expression level of *Mdfic2* in specific mouse (*C*) and human (*D*) tissues. (*E*) Mdfic2 expression in DRG neurons as detected by immunohistochemistry for Mdfic2 combined with neuronal markers in lumbar DRG sections. The top row shows that Mdfic2-positive neurons overlap largely with both nonpeptidergic nociceptors expressing isolectin B4 (IB4) and some peptidergic nociceptors expressing calcitonin gene-related peptide (Cgrp, bottom row). However, there is only a marginal overlap with large neurons immunoreactive for neurofilament-200 (NF200, middle row). Respective scale bars are shown in white.

We first identified *Mdfic2* (*Gm765*) by searching the mouse GeneAtlas GNF1M microarray dataset for genes with enriched expression in dorsal root and trigeminal ganglia tissue. Real-time quantitative PCR of cDNA tissue panels confirmed the enriched expression of *Mdfic2* in mouse and human DRG (Fig. 1 *C* and *D*). Analysis of a single-cell RNA sequencing (scRNA-seq) database of murine peripheral sensory neurons shows *Mdfic2* is expressed within *Mrgprd*+ nonpeptidergic neurons (PSNP2 and PSNP3) and a subset of *Cgrp*+ peptidergic neurons (PSPEP1) (Fig. 1*B*) (35, 36). Immunohistochemistry of adult wild-type DRG sections using custom rabbit polyclonal antibodies confirms Mdfic2 protein expression within specific subsets of IB4+ nonpeptidergic and *Cgrp*+ neurons (Fig. 1*E* and *SI Appendix*, Fig. S1). Importantly, scRNA-seq shows *Piezo2* to be coexpressed in *Mdfic2*+ somatosensory neurons, but with homologs *Mdfi* and *Mdfic* largely excluded from DRG (Fig. 1*B*) (35, 36, 39).

Using DRG RNA, we cloned the human *MDFIC2* gene, showing the existence of two splice variants (KC470081 (40) and KC470082 (41)) (Fig. 1*A*). *MDFIC2* is a 5 exon protein coding gene located within the gene footprint of the *SAMSSON* lncRNA. Open reading frame analysis predicts a smaller and larger isoform of 189 amino acids (AHA59118) and 202 amino acids (AHA59119), respectively. The mouse genome does not encode the alternatively spliced exon 3 that is found in humans. The human (AHA59118) and mouse proteins (NP_001121564) are highly conserved sharing 82% amino acid identity (*SI Appendix*, Fig. S2).

### Mdfi/Mdfic Interaction Site to Piezo Is Conserved in Mdfic2.

We next compared the amino acid sequences of Mdfic (NP_780297) and Mdfic2 (NP_001121564) proteins to see whether the main Piezo-interactive part of Mdfic is conserved in Mdfic2. Sequence similarity analyses show that, in particular, the C-terminal (Ct) α-helices show a high degree of conservation (53% identity, 63% similarity) (*SI Appendix*, Fig. S3*A*). Palmitoylation of cysteine amino acid residues in the MDFIC Ct α-helix is required for effective modulation of the PIEZO1 channel (30). Three cysteine residues in the Mdfic2-Ct α-helix (C170, C176, and C187) are conserved when compared to seven cysteines present in the Mdfic-Ct and face the same orientation in the C-terminal α-helix when the 3D models of these two helices are aligned. Moreover, the negatively charged amino acids (Asp and Glu) within these α-helices, such as D232 and E239 that potentially provide an interaction with positively charged amino acids of Piezo1 (30), are also conserved in Mdfic2 (*SI Appendix*, Fig. S3*A*).

We tested whether the larger model of Mdfic2 could be fitted into the cryoEM density map of Piezo1 complexed with Mdfic (30). An AlphaFold-made (42, 43) 3D model of Mdfic2 (*SI Appendix*, Fig. S3*B*) showed an α-helical structure made of seven α-helices for the C-terminal part of the protein starting from Ser79 and ending by Arg189 (110 C-terminal amino acids) connected by loops (*SI Appendix*, Fig. S3*B*). The first N-terminal 23 amino acids of Mdfic2 appear to form a long α-helix connected to the rest of the protein by an unstructured loop made of 55 amino acids (Dataset S1).

The three molecules of Mdfic2 (79 to 189) could be easily fitted into Piezo1 forming a complex, resembling three wedges between Piezo1 blades (*SI Appendix*, Fig. S3*C*). All three molecules of Mdfic2 were aligned with the respective C-terminal α-helices of Mdfic. The alignment showed sufficient space for Mdfic2 molecules to be positioned between the “blades” of the Piezo1 trimer without visible steric clashes (Movie S1). Altogether, this suggests that Mdfic2 could modulate PIEZO channels similarly to MDFI/MDFIC.

### Mdfic2 Modulation of Piezo1/2 Mediated Currents in a Heterologous System.

**Fig. 2.**
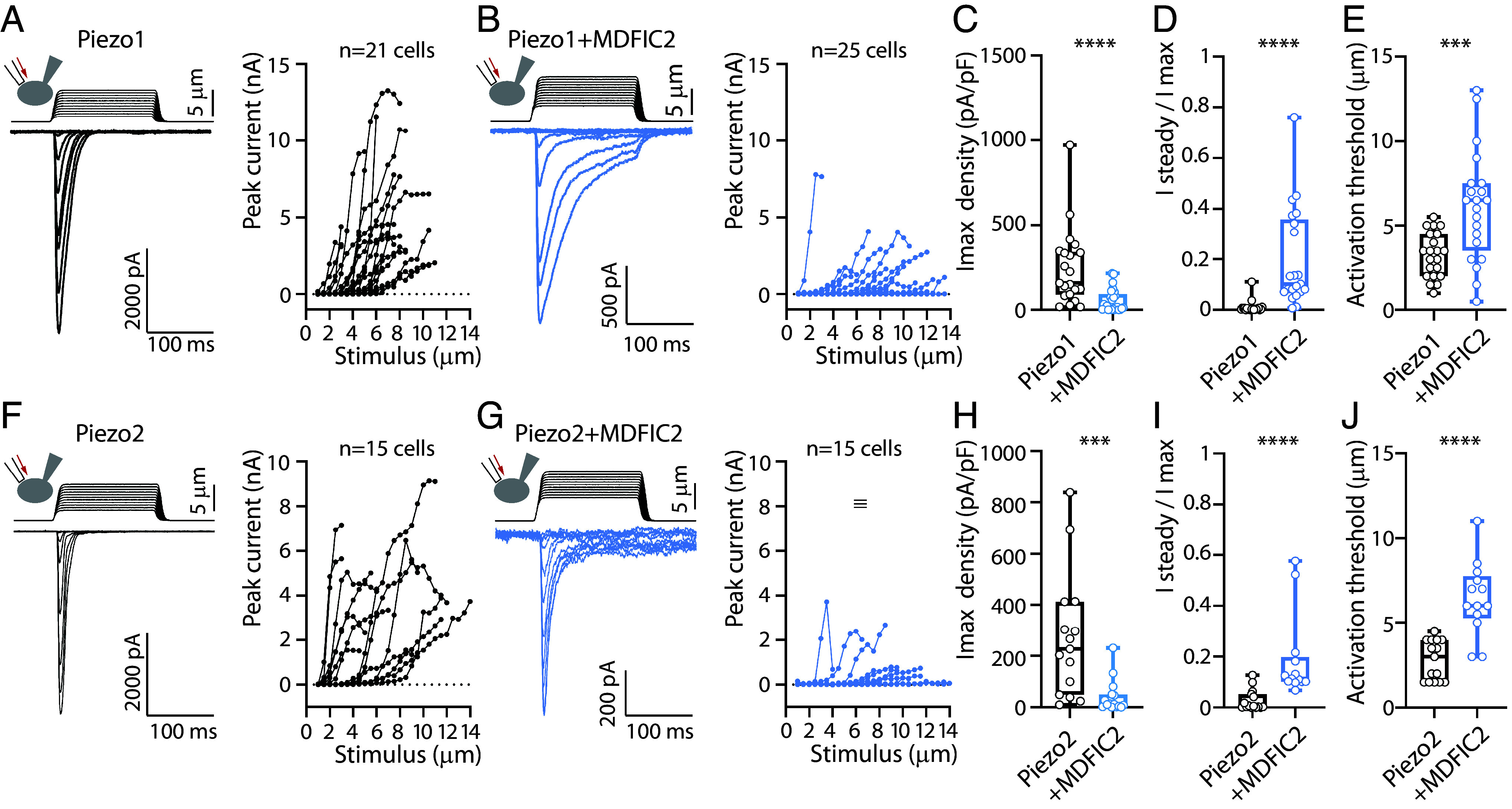
Mechano-clamp characterization of MDFIC2 modulation of Piezo1/2-mediated currents. (*A* and *B*) *Left* panels: Representative whole-cell recordings from HEK-P1KO cells transfected with Piezo1 (*A*) or Piezo1 + MDFIC2 (*B*), stimulated using a mechanical probe. *Right* panels: Current–stimulus relationships corresponding to the recordings shown in the *Left* panels. (*C*–*E*) Quantification of maximal current density amplitude (*C*), inactivation ratio (current remaining at the end of a 150 ms stimulation) (*D*), and mechanical activation threshold (*E*) in cells transfected with Piezo1 ± MDFIC2. (*F* and *G*) *Left* panels: Representative whole-cell recordings from HEK-P1KO cells transfected with Piezo2 (F) or Piezo2 + MDFIC2 (*G*), stimulated using a mechanical probe. *Right* panels: Current–stimulus relationships corresponding to the recordings shown in the *Left* panels. (*H*–*J*) Quantification of (*H*) maximal current density amplitude, (*I*) inactivation ratio, and (*J*) mechanical activation threshold in cells transfected with Piezo2 ± MDFIC2. Statistical significance: ***P < 0.001; ****P < 0.0001 (Mann–Whitney test). All recordings were conducted at a holding potential of Vh = −80 mV.

We next characterized the effect of MDFIC2 expression on Piezo1-mediated currents using whole-cell patch-clamp recordings in response to cell poking with a mechanical probe in HEK-P1KO cells (Fig. 2 *A*–*E*). Coexpression of MDFIC2 with Piezo1 led to a significant decrease in maximal amplitude (244.6 ± 49 pA/pF for control Piezo1 and 54.7 ± 13.2 pA/pF for Piezo1 + MDFIC2; mean ± SEM). This reduction in current amplitude was accompanied by a slowing of inactivation kinetics, as evidenced by a significant increase in the remaining current at the end of the 150 ms stimulus (1.0 ± 0.5% and 19.8 ± 4.2% of peak current amplitude for control Piezo1 and Piezo1 + MDFIC2, respectively). Additionally, the presence of MDFIC2 significantly increased the activation threshold (3.3 ± 0.3 µm for control Piezo1 and 6.1 ± 0.7 µm for Piezo1 + MDFIC2). Thus, MDFIC2 expression influences the amplitude, gating, and sensitivity of Piezo1-mediated currents. Similarly, MDFIC2 expression significantly modulated Piezo2-mediated currents (Fig. 2 *F*-*J*). Maximal amplitude was reduced (270.3 ± 62.6 pA/pF for control Piezo2 and 41.3 ± 16.6 pA/pF for Piezo2+ MDFIC2), while the ratio of remaining current at the end of mechanical stimulation increased (2.8 ± 1.0% and 18.9 ± 4.6% for Piezo2 alone and Piezo2 + MDFIC2, respectively). Furthermore, the activation threshold was significantly elevated in the presence of MDFIC2 (2.8 ± 0.3 µm and 6.4 ± 0.6 µm for Piezo2 alone and Piezo2 + MDFIC2, respectively). These findings highlight the similar regulatory effects of MDFIC2 on Piezo1- and Piezo2-mediated currents.

To further investigate the impact of MDFIC2 expression on mechanically activated Piezo currents, another mechanical stimulation paradigm was employed. In these experiments, pseudomacroscopic currents were recorded using negative pressure stimulation applied through the recording pipette in cell-attached configuration (*SI Appendix*, Fig. S4). These experiments focused on Piezo1-mediated currents, as Piezo2 has been reported to exhibit poor responsiveness to this stimulation protocol (44–46). Under these experimental conditions, the coexpression of MDFIC2 with Piezo1 strongly modulates channel gating. Piezo1-mediated currents elicited by a −80 mm Hg pressure step exhibit activation time constants of 16.29 ± 6.24 ms and 75.70 ± 27.70 ms (*SI Appendix*, Fig. S4*B*) and deactivation time constants of 27.96 ± 6.78 ms and 95.00 ± 47.96 ms (*SI Appendix*, Fig. S4*C*) without or with MDFIC2 coexpression, respectively. Control Piezo1 currents are characterized by an inactivation time constant of 61.14 ± 29.05 ms (n = 7 cells), whereas coexpression of MDFIC2 results in currents with minimal inactivation during stimulation, as indicated by the ratio of current remaining at the end of a 500 ms pressure step (0.30 ± 0.13 and 0.95 ± 0.07 for control Piezo1 and Piezo1 + MDFIC2, respectively) (*SI Appendix*, Fig. S4*D*). Next, the pressure sensitivity of Piezo1-mediated currents under negative pressure stimulation was characterized by determining the pressure required for half-maximal activation (P_50_). Since currents in the presence of MDFIC2 exhibit almost no inactivation, and to avoid contamination by channels not modulated by MDFIC2, pressure sensitivity in the presence of MDFIC2 was determined at the end of the stimulus (*SI Appendix*, Fig. S4 *E*–*H*). These results show that the P_50_ of Piezo1-mediated currents was significantly increased by approximately 10 mm Hg in the presence of MDFIC2 (*SI Appendix*, Fig. S4*I*). Finally, unitary current at −80 mV of Piezo1 channels was not statistically different when MDFIC2 was coexpressed (2.46 ± 0.08 and 2.402 ± 0.12 pA without and with MDFIC2, respectively) (*SI Appendix*, Fig. S4*J*). Therefore, although negative pressure stimulation in the cell-attached configuration and cell poking using a mechanical probe in the whole-cell configuration are not equivalent, both experiments demonstrate that the gating properties and mechanical sensitivity of Piezo-mediated currents are modified in the presence of MDFIC2.

### Mdfic2 Modulation of Mechanosensitive Currents in DRG Neurons.

**Fig. 3.**
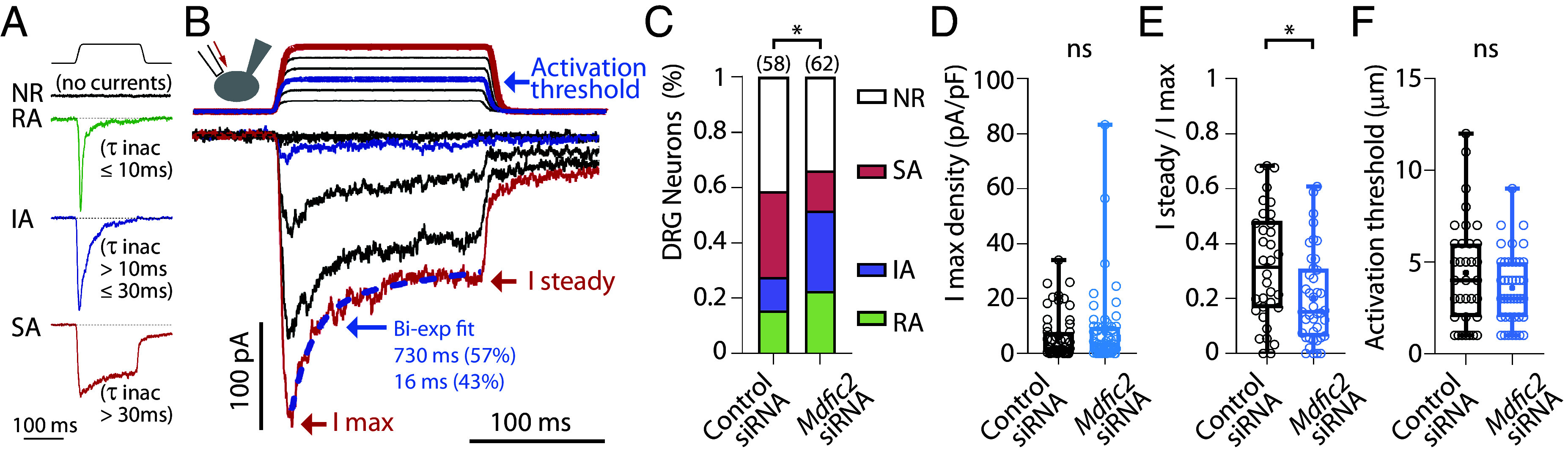
*Mdfic2* knockdown in nonpeptidergic nociceptors. (*A*) Representative traces of distinct current responses in DRG neurons following mechanical stimulation. These include rapidly adapting (RA), intermediately adapting (IA), and slowly adapting (SA) currents, classified based on their inactivation kinetics. NR: Nonresponsive neurons. (*B*) Example of superimposed traces showing incremental probe displacements (*Top*, 1 µm increments) and corresponding current recordings (*Bottom*) in a DRG neuron. The activation threshold (blue trace), maximal current (I max), and steady-state current (I steady) are indicated. Inactivation kinetics and the relative contributions of underlying mechanosensitive currents are determined using a biexponential fit (blue dotted line). (*C*) Distribution of mechanosensitive current types in IB4-positive neurons treated with either control or *Mdfic2* siRNA. For neurons exhibiting two current types, only the predominant one is considered. Statistical analysis: Chi-square test, *P <0.05. (*D*–*F*) Characterization of maximal current density amplitude (*D*), the ratio of current remaining at the end of the 150 ms stimulation (*E*), and the threshold of mechanical activation (*F*) in IB4-positive neurons treated with either control or *Mdfic2* siRNA, as specified. *P < 0.05, Mann–Whitney test. All recordings are made at Vh = −80 mV. For *E* and *F*, n = 34 and 41 neurons for control or *Mdfic2* siRNA, respectively.

We next performed small interfering RNA (siRNA) experiments in dorsal root ganglion neurons to determine whether Mdfic2 modulates mechanosensitive currents in sensory neurons. DRG neurons constitute a heterogeneous population and exhibit various mechanosensitive currents, which are typically classified into three groups based on their inactivation kinetics: rapidly, intermediately, and slowly adapting currents (RA, IA, and SA currents, respectively) (Fig. 3*A*) (1, 47, 48). These current types can be coexpressed within neurons, where fitting the inactivation kinetics using biexponential equations allows for the extraction of current types and their relative contributions (Fig. 3*B*), as done in a previous study (49). To identify transfected neurons, we coelectroporated siRNAs with a GFP-expressing plasmid, enabling fluorescent detection. A nontargeting siRNA was used as a negative control. Since expression data indicate that Mdfic2 is present in a subset of nonpeptidergic nociceptors (Fig. 1*B*), we selectively recorded from IB4^+^ neurons, identified using IB4-Alexa Fluor 568 conjugate staining (49). We then compared the proportions of DRG neurons classified according to their predominant mechanosensitive current component. Inhibition of Mdfic2 significantly altered the relative proportion of IB4^+^ DRG neurons exhibiting different MS currents (χ^2^ test, *P* = 0.0292), with a notable decrease in the incidence of SA current-expressing neurons from 31.0% to 14.5% and a corresponding increase in neurons expressing currents with faster inactivation kinetics (RA: 15.5 to 22.6%; IA: 12.1 to 29.0%) (Fig. 3*C*). Accordingly, the ratio of remaining current at the end of mechanical stimulation significantly decreased with *Mdfic2* knockdown (31.9 ± 3.5 to 20.1 ± 2.6, *P* = 0.013, Mann–Whitney test) while no differences were observed in maximal current amplitude and activation threshold (Fig. 3 *D*–*F*). The mild phenotype resulting from Mdfic2 inhibition on mechanosensitive currents in IB4^+^ DRG neurons may reflect the partial overlap of *Mdfic2* expression in IB4^+^ neurons, as well as the contribution of other PIEZO-independent, mechanically activated current types coexpressed in DRG neurons (49).

To further confirm the impact of Mdfic2 on Piezo-mediated currents in neurons, we leveraged the fact that NF low-threshold mechanoreceptors predominantly express Piezo2 currents (1, 14). This population of DRG neurons consists of large-diameter, IB4^−^ neurons (50). Since Mdfic2 is poorly expressed in these neurons (Fig. 1*B*), we electroporated *MDFIC2* IRES-mCherry expression plasmid into DRG neurons costained with IB4–FITC conjugate and recorded from large-diameter, IB4^−^ neurons.

**Fig. 4.**
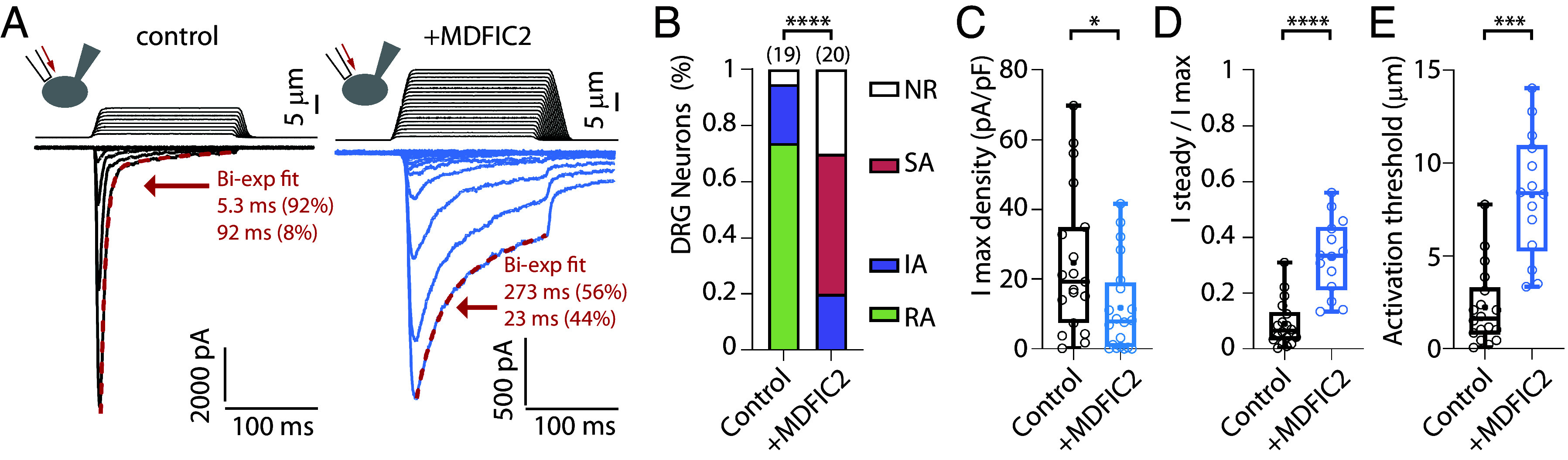
MDFIC2 overexpression in NF low-threshold mechanoreceptors. (*A*) Representative traces of current responses following mechanical stimulation in control (*Left* panels) and *MDFIC2*-transfected (*Right* panels) large-diameter IB4-negative DRG neurons. (*B*) Distribution of mechanosensitive current types in control or *MDFIC2-*transfected large-diameter IB4-negative neurons. For neurons exhibiting two current types, only the predominant one is considered. Statistical analysis: Chi-square test, ****P < 0.0001. (*C*–*E*) Characterization of maximal current density amplitude (*C*), the ratio of current remaining at the end of the 150 ms stimulation (*D*), and the threshold of mechanical activation (*E*) in control or *MDFIC2-*transfected large-diameter IB4-negative neurons. *P <0.05, ***P < 0.001; ****P < 0.0001; Mann–Whitney test. All recordings are made at Vh = −80 mV. For *D* and *E*, n = 18 and 14 neurons for control or *MDFIC2*, respectively.

As previously reported (49, 50), the majority (73.7%) of these neurons exhibit RA currents as their primary component under control conditions (Fig. 4 *A* and *B*). However, MDFIC2 expression significantly altered the distribution of DRG neurons based on their predominant mechanosensitive current component (χ^2^ test, *P* < 0.0001). Notably, in the presence of MDFIC2, half of the NF low-threshold mechanoreceptors exhibited SA currents as their main component, alongside a substantial increase in NR (nonresponding) neurons (from 5.3 to 30.0%) (Fig. 4 *A* and *B*). Accordingly, maximal current amplitude significantly decreased (24.7 ± 4.7 pA/pF to 11.7 ± 3 pA/pF, *P* = 0.024, Mann–Whitney test), while the ratio of remaining current at the end of mechanical stimulation significantly increased (8.9 ± 1.9% to 33.0 ± 3.6%, *P* < 0.0001, Mann–Whitney test) with MDFIC2 expression (Fig. 4 *C* and *D*). Similar to its effect on Piezo currents in HEK-P1KO cells, MDFIC2 expression in NF low-threshold mechanoreceptors also increased the activation threshold of mechanosensitive currents (4.8 ± 0.7 µm to 7.8 ± 0.6 µm, *P* = 0.001, Mann–Whitney test). Altogether, these results show MDFIC2 modulation of mechanosensitive currents in sensory neurons.

### Modulation of MDFIC2 in Naïve Mice Regulates Touch Sensitivity.

**Fig. 5.**
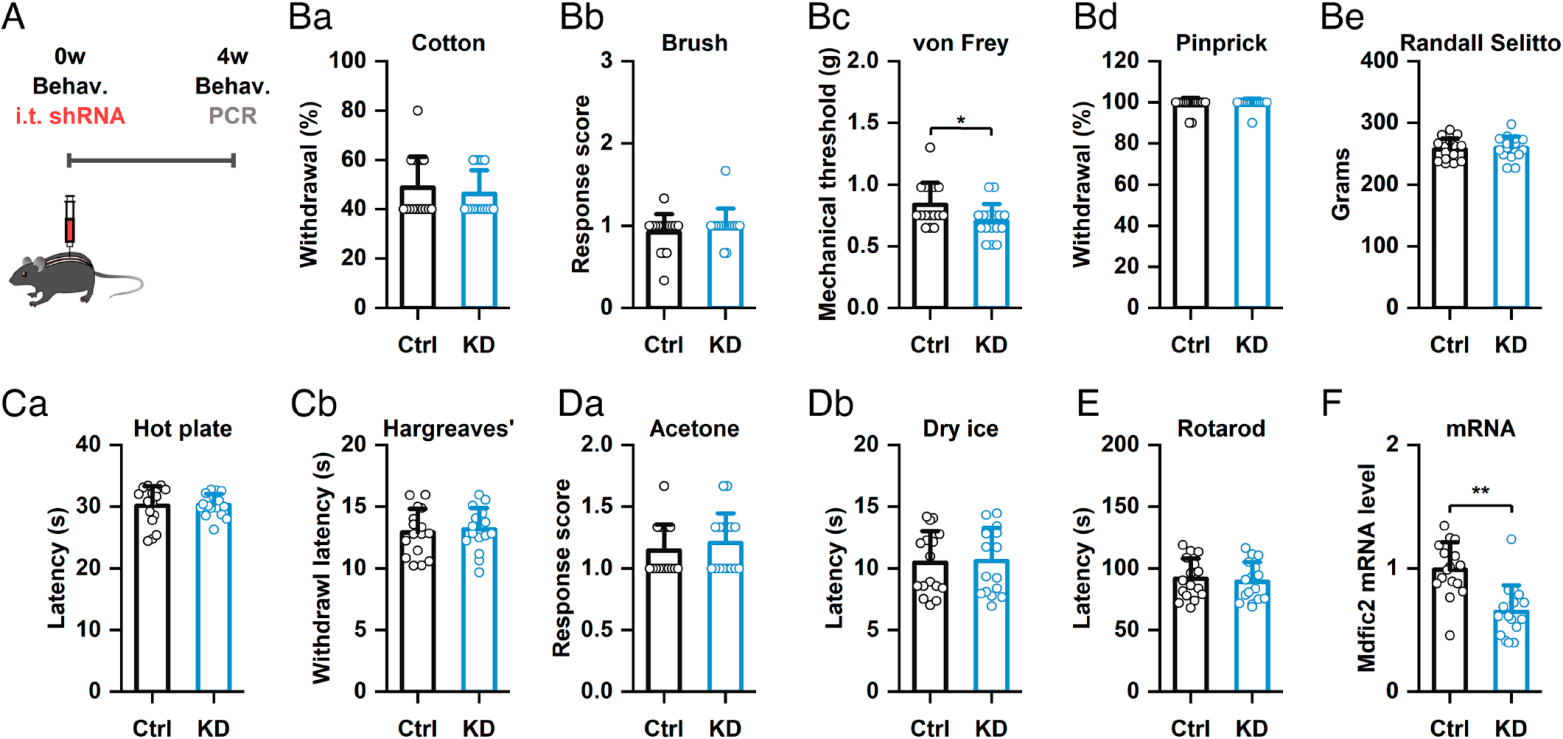
*Mdfic2* knockdown enhances mechanical sensitivity to von Frey filaments in naïve mice. (*A*) Detailed schematic representation of the experimental protocol. (*B*) Comprehensive analysis of *Mdfic2* knockdown effects on mechanical sensitivity, assessed through multiple modalities including cotton swab stimulation (*Ba*, unpaired *t* test, *P* = 0.53), brush application (Bb, unpaired *t* test, *P* = 0.29), von Frey filament testing (*Bc*, unpaired *t* test, *P = 0.02), pinprick examination (*Bd*, unpaired *t* test, *P* = 0.56), and pressure pain (Be, unpaired *t* test, *P* = 0.73) evaluation. (*C*) Investigation of *Mdfic2* knockdown effects on thermal nociception using hot plate (*Ca*, unpaired *t* test, *P* = 0.94) and radiant heat (*Cb*, unpaired *t* test, *P* = 0.69) assessments. (*D*) Examination of *Mdfic2* knockdown effects on cold sensitivity utilizing acetone application (*Da*, unpaired *t* test, *P* = 0.44) and cold plantar (*Db*, unpaired *t* test, *P* = 0.87) testing. (*E*) Evaluation of *Mdfic2* knockdown impact on motor function performance (unpaired *t* test, *P* = 0.68). (*F*) Quantitative real-time PCR validation of *Mdfic2* knockdown efficiency (unpaired *t* test, **P < 0.01). N = 16 mice for each group. Ctrl: control, KD: knockdown.

To investigate the physiological role of MDFIC2 in vivo, we tested the effects of *Mdfic2* knockdown in DRG four weeks after intrathecal administration of AAV-shRNA (Fig. 5 and *SI Appendix*, Fig. S9). Behavioral assessments revealed that *Mdfic2* knockdown mice exhibited no significant differences in mechanical sensation tests, including cotton swab, dynamic brush, pinprick, and Randall-Selitto, compared to controls. However, static von Frey testing demonstrated a modest but statistically significant reduction in mechanical thresholds in knockdown mice. Other behavioral parameters, including thermal sensitivity (Hargreaves’ and hot plate), cold responsiveness (acetone and cold plantar), and motor function (rotarod), remained unchanged between knockdown and control groups.

Next, we enhanced MDFIC2 expression levels in DRG through intrathecal administration of an AAV9 virus encoding CAG-*hMDFIC2*-IRES-mCherry (*SI Appendix*, Figs. S5 and S9). Four weeks later, behavioral assessments on test and control mice injected with a CAG-mCherry AAV9 were carried out. The cotton swab test revealed no significant differences in response frequency between overexpressing and control groups. However, *MDFIC2* overexpressing mice demonstrated significantly reduced scores in dynamic brush tests and elevated mechanical thresholds in static von Frey tests compared to controls. Notably, sensitivity to noxious mechanical stimuli, as assessed by pinprick and Randall-Selitto tests, remained comparable between groups. Thermal sensitivity, evaluated through hot plate and Hargreaves’ tests, showed similar latencies in both groups. Additionally, cold responsiveness, measured by acetone and cold plantar tests, exhibited no significant differences. Motor function, assessed via the rotarod test, remained unaffected by *MDFIC2* overexpression, with both groups displaying similar falling latencies. These findings collectively demonstrate that MDFIC2 plays a specific role in modulating tactile function in mice, particularly in regulating sensitivity to dynamic brush and static von Frey mechanical stimulation.

### *Mdfic2* Is Downregulated in Neuropathic Pain Models.

**Fig. 6.**
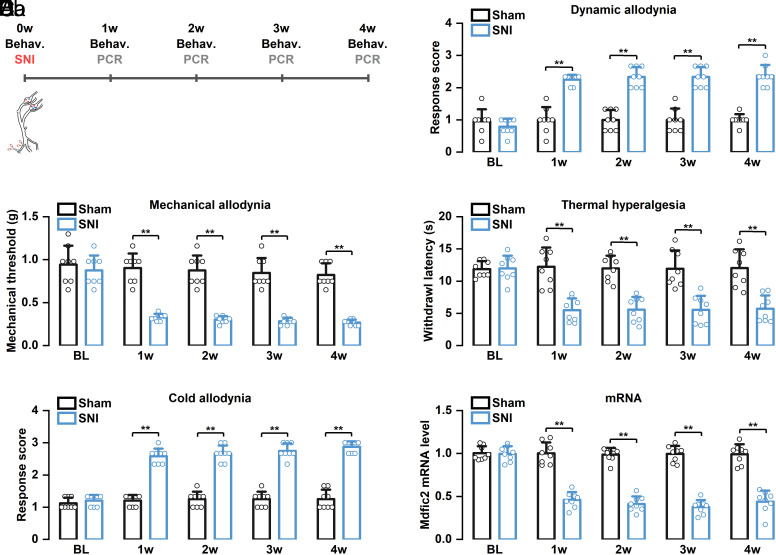
SNI induces the downregulation of *Mdfic2* mRNA expression in DRG neurons. (*A*) Schematic representation of the experimental design and timeline. (*B*) SNI procedure results in pronounced mechanical allodynia, characterized by both dynamic (*Ba*) and static (*Bb*) components. Two-way repeated-measures ANOVA, Bonferroni post hoc test, adjusted **P < 0.01. (*C*) SNI leads to enhanced thermal hyperalgesia, demonstrating increased sensitivity to heat stimuli. Two-way repeated-measures ANOVA, Bonferroni post hoc test, adjusted **P < 0.01. (*D*) SNI elicits significant cold allodynia, indicating heightened responsiveness to cold stimulation. Two-way repeated-measures ANOVA, Bonferroni post hoc test, adjusted **P < 0.01. (*E*) Quantitative analysis reveals a significant reduction in *Mdfic2* mRNA expression levels in DRG following SNI. Two-way ANOVA, Bonferroni post hoc test, adjusted **P < 0.01. N = 8 mice for each group. BL: baseline.

Mechanical allodynia, a hallmark symptom of neuropathic pain, involves PIEZO2 as a crucial molecular mediator (18, 22, 51–53). Given the modulatory role of MDFIC2 in PIEZO2 function, we investigated whether *Mdfic2* levels are regulated in the SNI neuropathic pain model (Fig. 6). SNI-operated mice exhibited pronounced mechanical allodynia, thermal hyperalgesia, and cold allodynia throughout the 4-wk postsurgical period. Weekly postsurgical DRG tissue analysis revealed significantly reduced *Mdfic2* mRNA expression in SNI mice compared to sham controls (Fig. 6*E*). This downregulation was replicated at the protein level as shown by immunofluorescent staining using a custom anti-Mdfic2 antibody (*SI Appendix*, Fig. S9*C*). To validate these findings, we established two additional neuropathic pain models: spinal nerve transection (SNT) and partial sciatic ligation (PSL). Both models demonstrated significant reduction in DRG *Mdfic2* mRNA expression (*SI Appendix*, Fig. S6). Furthermore, analysis of a DRG single-cell transcriptome sequencing dataset also revealed that SNT induced an approximately 8-fold reduction in *Mdfic2* mRNA levels (54). These findings collectively demonstrate that nerve injury significantly downregulates *Mdfic2* expression in DRG, suggesting its potential role in neuropathic pain pathogenesis and progression.

We examined the effects of *Mdfic2* knockdown on neuropathic pain development and maintenance. Intrathecal administration of AAV-shRNA 1 wk after SNI surgery effectively reduced *Mdfic2* mRNA expression in DRG but did not affect established mechanical allodynia or other pain-related behaviors (*SI Appendix*, Fig. S7). Specifically, dynamic brush and static von Frey assessments revealed comparable scores and mechanical thresholds between *Mdfic2* knockdown and control groups from weeks 2 to 5 postsurgery. Similarly, thermal sensitivity and cold responsiveness, evaluated through Hargreaves’ and acetone tests, respectively, showed no significant differences between groups. We also tested the effects of *Mdfic2* knockdown via intrathecal AAV-shRNA administration three weeks prior to SNI surgery. Subsequent behavioral analyses demonstrated that *Mdfic2* knockdown did not significantly influence SNI-induced pain behaviors (*SI Appendix*, Fig. S8). Hence, neither prophylactic nor therapeutic knockdown of *Mdfic2* substantially influences the development or maintenance of neuropathic pain.

### *MDFIC2* Overexpression Reduces Mechanical Allodynia.

**Fig. 7.**
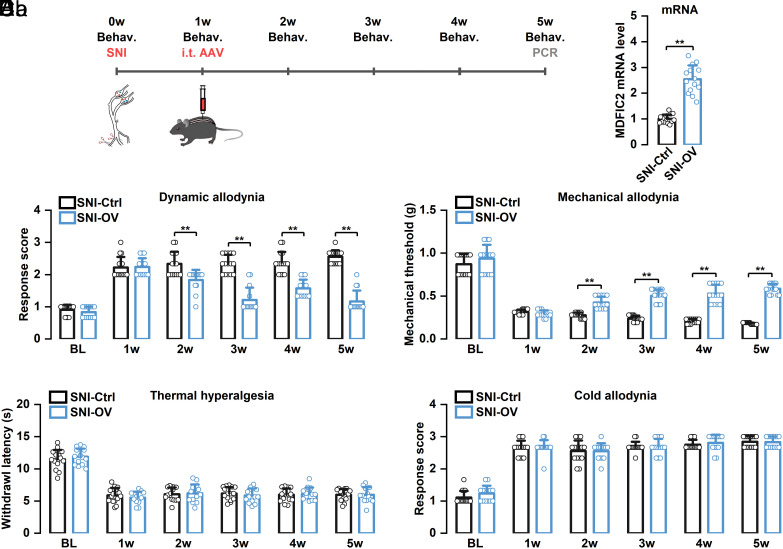
*MDFIC2* overexpression suppressed established mechanical hypersensitivity in SNI mice. (*A*) Schematic illustration showing the timeline of AAV-*MDFIC2* injection, SNI surgery, and behavioral testing protocol. (*B*) RT-qPCR measurements demonstrating successful *MDFIC2* upregulation (unpaired *t* test, **P < 0.01). (*C*) AAV-mediated *MDFIC2* overexpression attenuated both dynamic (*Ca*) and static (*Cb*) mechanical hypersensitivity following SNI. Two-way repeated-measures ANOVA, Bonferroni post hoc test, adjusted **P < 0.01. (*D*) Elevated *MDFIC2* levels via AAV-*MDFIC2* delivery showed no effect on thermal hyperalgesia after SNI. Two-way repeated-measures ANOVA, Bonferroni post hoc test, adjusted *P* > 0.05. (*E*) Cold sensitivity post-SNI remained unchanged by AAV-*MDFIC2* treatment after SNI. Two-way repeated-measures ANOVA, Bonferroni post hoc test, adjusted *P* > 0.05. N = 15 and 14 mice for each group. Ctrl: control, OV = overexpression, BL: baseline.

To explore whether regulating *MDFIC2* expression could modulate mechanical allodynia in neuropathic pain, we first intrathecally administered AAV-*MDFIC2* one week post–SNI surgery in mice that had already developed mechanical allodynia. Our findings demonstrated that, compared to control mice, intrathecal administration of AAV-*MDFIC2* significantly elevated DRG *MDFIC2* mRNA expression levels and markedly attenuated mechanical allodynia in SNI mice (Fig. 7). Specifically, in dynamic brush and static von Frey assessments, *MDFIC2*-overexpressing mice exhibited significantly reduced response scores and elevated mechanical thresholds from weeks 2 to 5 postsurgery compared to control mice. Notably, *MDFIC2* overexpression did not influence thermal hyperalgesia or cold allodynia in SNI mice. In Hargreaves’ and acetone assessments, no significant differences were observed in thermal latency and cold response scores between *MDFIC2*-overexpressing and control groups from weeks 2 to 5 postsurgery. Furthermore, no significant differences were observed in spontaneous pain behaviors under baseline SNI conditions, nor after administration of the α1-adrenergic receptor agonist and vasoconstrictor, phenylephrine, which is used to enhance spontaneous behaviors through modulation of DRG vascular dynamics (*SI Appendix*, Fig. S10) (55).

**Fig. 8.**
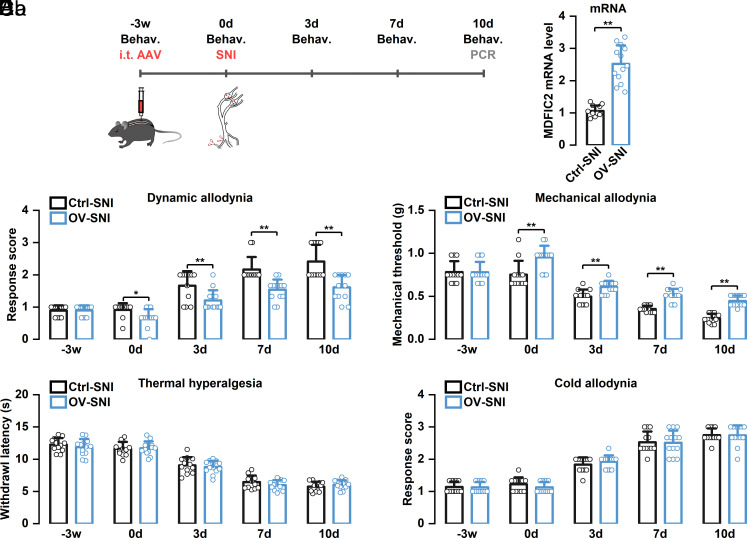
Pretreatment with *MDFIC2* overexpression inhibited mechanical allodynia in SNI mice. (*A*) Schematic representation of experimental timeline depicting AAV delivery, SNI procedure, and behavioral evaluation sequence. (*B*) RT-qPCR analysis validating effective *MDFIC2* upregulation (unpaired *t* test, **P < 0.01). (*C*) Introduction of AAV-*MDFIC2* reduced dynamic (*Ca*) and static (*Cb*) mechanical allodynia post-SNI. Two-way repeated-measures ANOVA, Bonferroni post hoc test, adjusted *P = 0.016, **P < 0.01. (*D*) Elevated *MDFIC2* expression through AAV delivery did not block the manifestation of heat hypersensitivity following SNI. Two-way repeated-measures ANOVA, Bonferroni post hoc test, adjusted *P* > 0.05. (*E*) Cold sensitivity development after SNI was not affected by AAV-*MDFIC2* administration. Two-way repeated-measures ANOVA, Bonferroni post hoc test, adjusted *P* > 0.05. N = 12 and 13 mice for each group. Ctrl: control, OV: overexpression.

Next, to investigate the impact of *MDFIC2* overexpression on the development of neuropathic pain-induced mechanical allodynia, we administered AAV-MDFIC2 intrathecally 3 wk prior to SNI surgery. Behavioral assessments revealed that, consistent with naive mice, *MDFIC2* overexpression itself reduced dynamic brush test scores and elevated static von Frey test mechanical thresholds before surgery (Fig. 8). Moreover, *MDFIC2* overexpression significantly attenuated mechanical allodynia at days 3, 7, and 10 post–SNI surgery, as demonstrated by reduced dynamic brush test response scores and increased static von Frey test mechanical thresholds. However, *MDFIC2* overexpression did not alter the temporal progression of neuropathic pain-induced mechanical allodynia; similar to the control group, *MDFIC2*-overexpressing mice reached peak mechanical allodynia at day 10 post–SNI surgery. These findings demonstrate that *MDFIC2* overexpression can effectively ameliorate neuropathic pain-induced mechanical allodynia, highlighting its potential therapeutic value in neuropathic pain management.

## Discussion

A recent study identified MDFI and MDFIC as potent modulators of PIEZO1 and PIEZO2 channels (30), suggesting they could play significant roles in various biological functions through their interactions with PIEZOs. Our findings demonstrate that MDFIC2, like MDFI and MDFIC, modulates the biophysical properties of both PIEZO1 and PIEZO2 channels. We used two methods to characterize PIEZO currents, cell-poking and stretch experiments, that are complementary but not equivalent (for review see ref. 56). Coexpression experiments revealed that MDFIC2 alters the kinetics of PIEZO currents in cell-poking experiments (increase in current remaining at the end of the mechanical stimulation) and in stretch experiments (no apparent inactivation, slowing of activation, and deactivation) similarly to the modulation of PIEZO currents by MDFI and MDFIC (30). Moreover, MDFIC2 affects the mechanical sensitivity of PIEZO channels by increasing the activation threshold in cell-poking experiments and by an approximately 10 mmHg rightward shift of the PIEZO1 P_50_ value determined in stretch experiments. A similar shift in pressure sensitivity has been described for MDFIC and PIEZO1 (30). Altogether, this suggests that MDFIC2 interacts with PIEZO channels in a manner similar to what was demonstrated for MDFIC and PIEZO1 by cryoelectron microscopy (30). In line with this, AlphaFold predictions and PyMOL 3D modeling show that the C-terminal α-helix of Mdfic2 aligns with that of Mdfic bound to Piezo1 (*SI Appendix*, Fig. S3), suggesting that functional changes are caused by this direct interaction. However, since MDFI and MDFIC are known to bind transcription factors (33, 57) and to AXIN1 (58), we cannot rule out the possibility that MDFIC2 expression triggers cellular responses that influence PIEZO channel activity. For example, MDFIC has been shown to regulate β1-integrin activation in lymphatic endothelial cells, influencing cellular adhesion to extracellular matrix components (33), processes that could influence plasma membrane tension (59), and ultimately affect PIEZO activity. Therefore, MDFIC2 may modulate PIEZO channel activity either directly, by interacting with the channels or functioning as a scaffolding subunit that links them to other partners, or indirectly, through the regulation of gene expression that affects membrane lipid composition, cytoskeletal dynamics, or extracellular matrix organization. Although no function has been reported so far regarding MDFIC2, further studies will be needed to explore these possibilities and fully understand the mechanism(s) by which MDFIC2 modulates PIEZO channel function. An important focus will be to compare the gene expression changes downstream of PIEZO channel activity in cells deficient of and overexpressing *Mdfic2*.

What could be the consequences of MDFIC2 expression on PIEZO channel signaling? Slowing inactivation can increase signaling, as shown for human “gain-of-function” PIEZO1 and PIEZO2 mutations leading to dehydrated hereditary stomatocytosis (60) and distal arthrogryposis (61), respectively. However, this is only valid if pressure sensitivity is constant (then for a given stimulation, more ions pass through the channel). On the contrary, our results show that coexpression of MDFIC2 with Piezo channels induces a reduction in maximal current amplitude in cell-poking experiments, suggesting decreased signaling. This effect is likely due to the MDFIC2-induced decrease in Piezo1 and Piezo2 pressure sensitivity, as patch rupture occurring at high stimulation intensities can lead to an underestimate of maximal current amplitude when MDFIC2 is expressed. This is supported by the rare instances where a plateau current is reached with MDFIC2 compared to Piezos alone (Fig. 2). Importantly, although stretch experiments require particular attention to evaluate channel density (56), no obvious effect on pseudomacroscopic current amplitude was observed using this technique (*SI Appendix*, Fig. S4 *F* and *H*), where triaging led to the analysis of recording patches in which channel activity reached a plateau. This aligns with the original study showing that MDFIC modulates PIEZO1 properties without altering channel expression (30). Therefore, the MDFIC2-induced decrease in pressure sensitivity is expected to have the most relevant impact by reducing PIEZO channel signalization for stimulations in the physiological range.

In DRG neurons, *Mdfic2* mRNA is predominantly expressed in Mrgprd^+^ neurons, one of the primary populations of polymodal C-fiber nonpeptidergic neurons that mainly innervate the skin (62, 63). We conducted siRNA experiments in IB4^+^ neurons, which constitute a subpopulation of DRG neurons that includes virtually all Mrgprd^+^ neurons, accounting for approximately 75% of IB4^+^ neurons (62). In this set of experiments, no significant effect was observed regarding maximal current amplitude or activation threshold. However, IB4^+^ neurons express a mixture of mechanosensitive ion channels (49), including yet unknown channels distinct from PIEZOs. Therefore, the presence of channels that are not modulated by Mdfic2 is likely masking the impact of *Mdfic2* knockdown on PIEZO channels in these neurons. Although no effect on activation threshold was detected, *Mdfic2* siRNA induces a significant change in inactivation kinetics of mechanosensitive currents, consistent with the effect of MDFIC2 on PIEZO channels. In line with these in vitro observations, behavioral experiments performed using shRNA-induced *Mdfic2* knockdown show a slight mechanosensory phenotype, characterized by a decrease of von Frey mechanical threshold. These results suggest, together with the remarkable reduction of *Mdfic2* expression levels in three distinct models of neuropathic pain (SNT, PSL, and SNI), that *Mdfic2* down-regulation could contribute to chronic mechanical pain hypersensitivity. Whether this downregulation is due to reduced RNA stability, protein degradation, and/or decreased transcription remains to be determined. Interestingly, our shRNA knockdown approach failed to link a decrease in *Mdfic2* expression to painful mechanical sensitivity. Moreover, we did not explore the involvement of Mdfic2 in mechanical itch-related behavior, a process involving Piezo1 (19). Future studies using more specific approaches, including genetically modified animals, are required to determine the exact role of Mdfic2 to the various mechanosensory modalities under physiological and pathological conditions. For example, given the importance of PIEZO2 in somatosensory neurons for controlling gastrointestinal transit, it will be interesting to explore the role of MDFIC2 in mechanosensory gut regulation (64).

We tested the impact of *MDFIC2* cDNA expression in low-threshold mechanoreceptors that do not express *Mdfic2* but display predominantly Piezo2 mechanosensitive currents (14). This set of experiments recapitulates the results of coexpression experiments in HEK-P1KO cells, namely potent slowing of inactivation kinetics as well as increase in activation threshold of mechanosensitive currents. These results demonstrate the ability of MDFIC2 to modulate Piezo currents in sensory neurons in vitro. Consistently, intrathecal administration of AAV vector encoding *MDFIC2* cDNA reduces innocuous mechanical sensitivity to dynamic brush and static von Frey assays. We performed a battery of behavioral tests showing that this effect is specific for mechanosensation, in agreement with the phenotype of Piezo2 knock-out animals (14, 51). Although PIEZO2 has been shown to partially contribute to mechanical pain under physiological conditions (51), AAV-driven *MDFIC2* expression did not change mouse sensitivity to the pinprick and Randall Selitto mechanical pain tests. This could reflect that expressing *MDFIC2* in neurons already expressing it, such as those involved in mechanical pain response, has no effect.

Finally, we demonstrate that inducing the expression of *MDFIC2* in DRG neurons counteracts the mechanical allodynia that develops in the SNI model. This beneficial effect is observed when *MDFIC2* expression is induced either before or after the development of chronic pain. Notably, *MDFIC2* expression has no effect on thermal hypersensitivity, further confirming its specificity for mechanosensation. Given that Piezo2 is involved in mechanical allodynia (51), it may be hypothesized that *MDFIC2* expression induces a shift in Piezo2 mechanical sensitivity, thereby alleviating mechanical pain hypersensitivity. However, our study is limited by the absence of experiments using Piezo2 knockout mice, and thus, the possibility that MDFIC2 may also affect other mechanically activated channels cannot be excluded. Future work will explore whether MDFIC2 expression can reduce mechanical pain sensitivity in other chronic pain conditions, in particular in osteoarthritic mechanical pain where Piezo2 is also important (52).

Our results suggest that the MDFIC2-PIEZO binding represents a promising therapeutic pathway that warrants further exploration. Future studies investigating peptides or synthetic molecules capable of binding the pore module of PIEZO channels similarly to the C-terminal alpha helices of MDFI/MDFIC/MDFIC2 may provide therapeutic options for treating PIEZO-related disorders. This will need a clearer understanding of the likely posttranslational palmitoylation mechanism at the C terminus of MDFIC2. Additionally, genetic approaches to fine-tune delivery of *MDFIC2* to specific PIEZO2-expressing neurons may also be therapeutically beneficial. Outside of the DRG, *Mdfic2* is known to be a marker for specific vagal sensory neurons that innervate the esophagus and stomach (39). By exploring the tissue and developmental expression profile of *Mdfic2*, we will be able to better understand the potential contribution of Mdfic2 to the range of mechanical interoceptive processes that Piezo2 also regulates (65). Considering PIEZO channels are integral to maintaining homeostasis in various physiological processes, our findings open broad avenues with implications for numerous pathological conditions, in particular the mechanical allodynia suffered by patients with neuropathic pain, cancer, osteoarthritis, and/or gastrointestinal disorders such as irritable bowel syndrome.

## Materials and Methods

### Human MDFIC2 Cloning.

Mouse *Gm765* guided BLAT identification of human *MDFIC2* on chromosome 3, and human DRG cDNA was amplified, cloned, and sequenced, yielding two splice variants (KC470081, KC470082). The longer isoform (KC470082) was HA-tagged and cloned into CAG-h*MDFIC2*-IRES-mCherry alongside a CAG-mCherry control.

### HEK-P1KO Transfection.

PIEZO1-deficient HEK293T cells were cultured in DMEM + FBS (10%)-penicillin/streptomycin (1%), plated on poly-D-lysine coverslips, and transfected (Lipofectamine 3000) with mPiezo1/2-IRES-GFP plus mCherry or hMDFIC2-IRES-mCherry. GFP/mCherry-positive cells were analyzed 48 h later.

### Animals.

Adult C57BL/6 mice (8 to 12 wk, both sexes) were group-housed with food/water ad libitum under a 12:12 light–dark cycle. Mice were randomized, experiments performed blind, and sample sizes based on prior studies. All procedures were approved by Zhejiang IACUC (Protocol IACUC-20241119001) and, in the United Kingdom, conducted under the Animals (Scientific Procedures) Act 1986 (PPL 70/7382).

### Viral Vectors.

AAV9 vectors (VectorBuilder) included CAG-h*MDFIC2*-IRES-mCherry with control, and CAG-mCherry-U6-m*Mdfic2*_shRNA (target: GCAGACGAGAAACCTATTAAT) with scramble control. Viruses were produced in HEK293 cells, purified by ultracentrifugation, and yielded titers >1 × 10^13^ GC/ml.

### Data.

The mouse GeneAtlas MOE430 gcrma microarray data were accessed at https://biogps.org/. Single-cell RNA sequencing data were accessed at https://github.com/linnarsson-lab/adolescent-mouse, https://painseq.webflow.io/, and http://mousebrain.org/. AlphaFold was accessed at https://alphafold.ebi.ac.uk/ and PyMOL version 3.04 (PyMOL Molecular Graphics System) at https://www.pymol.org/.

A detailed description of materials and methods is provided in *SI Appendix*, *Materials and Methods*.

## Supplementary Material

Appendix 01 (PDF)

Dataset S01 (TXT)

Movie S1. Three molecules of Mdfic2 ((Ser79-Arg189) in cyan hues, AlphaFold models) are fitted into EM 3D structure of Piezo1 (in slate blue, PDB: 8imz) complexed with three C-terminal α-helices of Mdfic (in magenta). All three molecules of Mdfic2 are aligned with the respective C-terminal α-helices of Mdfic.

## Data Availability

MDFIC2 mRNA sequences are deposited in GenBank (40, 41). All study data are included in the article and/or supporting information.
