# Supplementary material for: MDFIC2 is a PIEZO channel modulator that can alleviate mechanical allodynia associated with neuropathic pain
Source: Proc Natl Acad Sci U S A. 2025 Nov 7;122(45):e2512426122. doi: 10.1073/pnas.2512426122 (PMC12626003; doi:10.1073/pnas.2512426122)
Supplement: Supplementary file 1 — Appendix 01 (PDF) [file pnas.2512426122.sapp.pdf]

This PDF file includes:

Supplementary text

Supplementary Figures: Fig S1-S10

Legends for Movie S1

Other supporting materials for this manuscript include the following:

Video S1

Dataset

Supplementary text

Materials and Methods

Cloning of human *MDFIC2*

Using the Blat tool (Human BLAT Search (ucsc.edu)) we queried the human genome with the mouse *Gm765* gene sequence (BC147266). A syntenic region on human chromosome 3 was identified with homology to a single EST (DR731266) mapping to the 3' end of *MDFIC2*. Predicted splice donor and acceptor sites were annotated and primers designed. Human DRG RNA (Clontech) was converted to cDNA and overlapping gene fragments spanning the entire open reading frame were amplified using primers 5'-TGG ACA TCC AGA GAC AAG GGA G and 5'-ATT TTG GTA CAC ACC GTT TCG CAG; 5'-ATG TCA GAA ACT GAG CTG GAA AAG AT and 5'-CTA GCG GTA ACA GAT TTC TGA AAT C. Amplicons were cloned into pCR-Blunt II-TOPO and Sanger sequenced. Two splice variants were identified and deposited into GenBank under accession numbers KC470081 and KC470082. The longest splice variant (KC470082) was HA-tagged at the N terminus and cloned into a mammalian expression vector upstream of IRES-mCherry (CAG-h*MDFIC2*-IRES-mCherry) (VectorBuilder) alongside a CAG-mCherry control construct.

Real-time quantitative PCR of tissue panels

Mouse and human RNA samples were purchased from Clontech, with the exception of mouse hippocampus, amygdala and DRG which were dissected in-house from adult C57BL/6 mice and RNA isolated using the PureLink™ RNA Micro Scale Kit (Invitrogen) according to the manufacturer's procedures. Reverse transcription was performed using iScript cDNA synthesis kit (BioRad). TaqMan real-time PCR (ThermoFisher) was carried out using the following probes for mouse *Mdfic2* (Mm01182008_m1) and *Actb* (Mm01205647_g1). Expression levels in human tissues were determined using the SSOAdvanced Universal SYBR Green Supermix (Bio-Rad) and primers for human *MDFIC2* (h*MDFIC2*), Forward: 5'-GACTTCAATATCACAGATGGACCAG-3', Reverse: 5'-ATTTTGGTACACACCGTTTCGCAG-3'; human Actin (*ACTB*), Forward: 5'- CACCATTGGCAATGAGCGGTTC-3', Reverse: 5'-AGGTCTTTGCGGATGTCCACGT-3'. The expression level of target genes was normalized to the housekeeping β-actin gene mRNA. Relative gene expression [relative quantities (RQ) value] was determined using the $2^{-\Delta\Delta Ct}$ equation in which the expression level in DRG was used as the calibrator.

Immunohistochemistry

Mouse DRG sections were obtained from adult wild type C57BL/6 mice. DRGs were extracted from the lumbar area and fixed with 4% paraformaldehyde (PFA) in PBS (pH 7.4) for 2 h at 4 °C before being embedded in cryopreservative solution (30% sucrose) overnight at 4°C. Tissue samples were then placed in OCT blocks for posterior sectioning by cryostat. Sections (~11 µm thick) were mounted onto Superfrost Plus (Fisher Scientific) slides, allowed to freeze-dry overnight at −80 °C, for an immediate use, or were stored at −80°C in air-tight containers for no longer than a month for subsequent experiments.

For immunohistochemistry, DRG sections were removed from −80°C, rinsed with PBS (pH 7.4), fixed with 4% PFA in PBS (pH 7.4) for 15 minutes at room temperature, washed three times with PBS, permeabilized in 0.3% Triton X-100 in PBS (pH 7.4) for 15 minutes at +4°C, washed three times with PBST (0.01% Triton X-100 in PBS pH 7.4), blocked with 5% BSA in 0.1% Triton X-100 in PBS pH 7.4 for one hour at room temperature and incubated with primary antibodies overnight at +4°C. Primary antibodies were mouse anti-Neurofilament 200 (NF200; Sigma N0142 clone N52, 1:400); goat anti-calcitonin-gene-related peptide (Cgrp; Abcam ab-36001, 1:1000) and two custom rabbit anti-Mdfic2 antibodies (at 1:100 and 1:200). The antibodies to mouse Mdfic2 were raised in 2 host rabbits after immunisation with peptides ELEKIKVRTAEH and GPAQETPNEKNLSGS (Eurogentec). Isolectin B4 (IB4) binding was visualized using biotin-conjugated IB4 (Sigma, L2140, 1:1000) and Streptavidin conjugated with AF488 (Invitrogen S32354, 1:1000). Samples were then washed four times with PBST (0.01% Triton X-100 in PBS pH 7.4) and incubated for 1h at room temperature with secondary antibodies diluted in 0.1% Triton X-100 in PBS pH 7.4. Secondary antibodies were goat anti-mouse Alexa Fluor 488 (Invitrogen A11029), chicken anti-rabbit Alexa Fluor 594 (Invitrogen A21442) and chicken anti-goat Alexa Fluor 488 (Invitrogen A21487) all at 1:1000. Samples were washed four times with PBST (0.01% Triton X-100 in PBS pH 7.4) and once with PBS, air-dried briefly at room temperature and mounted with Vectashield mounting medium containing DAPI (H-1200).

Fluorescence was detected using Zeiss LSM 880 microscope. Images exported as 16-bit uncompressed tiff files for further basic editing in Adobe Lightroom Classic (Adobe) on a colour calibrated iMac (X-Rite) 5k retina monitor. Final images were exported as jpeg files with 6400 pix on the longest side at 300 ppi.

HEK-P1KO cell culture and transfection

PIEZO1-deficient HEK293T cells (1) were grown in Dulbecco's Modified Eagle Medium containing 4.5 mg.ml^{-1} glucose, 10% (vol/vol) FBS, 100 U.ml^{-1} (1%) penicillin/streptomycin. Cells were plated onto poly-D-lysine-coated 12-mm round glass coverslips (Corning) in 24-well plates and transfected using lipofectamine 3000 (Invitrogen) according to the manufacturer's

instruction. Each well was transfected with 300 ng.ml^{-1} of mPiezo1 IRES-GFP or mPiezo2 IRES-GFP construct together with 600 ng.ml^{-1} mCherry (control) or hMDFIC2 IRES-mCherry construct. GFP/mCherry-positive cells were observed and recorded 48 hours after transfection.

Dorsal root ganglion neuron culture and electroporation

Mice were terminally anaesthetized with isoflurane and euthanized by decapitation. DRG were isolated and incubated in enzyme solution containing 2 mg.ml^{-1} of collagenase 1A (Sigma-Aldrich) and 5 mg.ml^{-1} of dispase II (ThermoFisher) for 45 min at 37°C (2). The tissue was washed several times and triturated in 1 ml Hanks' balanced salt solution (HBSS, Gibco). 1 ml Bovine Serum Albumin (BSA, 15%, Sigma-Aldrich) was carefully added at the bottom of the resulting cell suspension and centrifuged (300×g for 5 min). The pellet was rinsed with Dulbecco's Phosphate Buffered Saline (DPBS, Gibco) and centrifuged (300×g for 2 min). The cell pellet was resuspended in Neon® Transfection System resuspension Buffer R (Invitrogen) and gently mixed with either 30 ng.µl^{-1} of pIRES2-AcGFP1 plasmid (Aequorea coerulescens GFP, Clontech; referred in results as GFP-plasmid) and 250 nM siRNA pool, or with 30 ng.µl^{-1} hMDFIC2-IRES-mCherry or mCherry (control) plasmids. Ten microliters of the cell suspension were electroporated using the Neon® Transfection System (Invitrogen) according to the manufacturer's protocol with the following program: 1200 V, 2 pulses, 20 ms. Electroporated cells were plated onto poly-D-lysine-coated 12-mm round glass coverslips (Corning) in 24-well plates in antibiotics-free media. Two hours later, the medium was replaced by Dulbecco's modified Eagle's medium (DMEM, High glucose, pyruvate, ThermoFisher) supplemented with 10% heat-inactivated Fetal Bovine Serum (FBS, Gibco), 100 U.ml^{-1} (1%) penicillin–streptomycin (Gibco), 100 ng.ml^{-1} nerve growth factor (NGF, Merck Millipore), and 50 ng.ml^{-1} glial-derived neurotrophic factor (GDNF, ThermoFisher). Media were changed 48 hours later.

Pools of 4 siRNA targeting *Mdfic2*, or non-targeting siRNA for control experiments were purchased at Horizon Discovery (#L-056902-01-0005 and #D-001810-10-05 for *Mdfic2* and control siRNA, respectively).

Transfected DRG neurons were visualized by the expression of GFP or mCherry. Cells were recorded at 3-4 days *in vitro* (DIV) following siRNA electroporation and 2-3 DIV following cDNA electroporation. Neurons were incubated with 1 µg.ml^{-1} isolectin GS-IB4 from Griffonia simplicifolia, AlexaFluor 568- or FITC-conjugate (Invitrogen) at 37°C for 10 minutes before recording.

Mechano-clamp experiments

Patch-clamp experiments were performed under whole-cell configuration using an Axopatch 200B amplifier (Axon Instruments) (2). Patch pipettes had resistances of 2-3 MΩ when filled with an internal solution consisting of (in mM) 140 CsCl, 10 Hepes, 5 EGTA, 1 $CaCl_2$, 1 $MgCl_2$, 4 MgATP and 0.4 Na_2GTP (pH adjusted to 7.3 with CsOH). The extracellular solution consisted of (in mM) 133 NaCl, 3 KCl, 1 $MgCl_2$, 10 Hepes, 2.5 $CaCl_2$, 10 glucose (pH adjusted to 7.3 with NaOH). All experiments were done at room temperature. Currents were sampled at 20 kHz and filtered at 2 kHz.

Mechanical stimulation was achieved using a fire polished glass pipette (tip diameter 3-4 µm) positioned at an angle of 80° and in contact with the cell being recorded. Downward movement of the probe toward the cell was driven by a Clampex controlled piezo-electric crystal microstage (E625 LVPZT Controller/Amplifier, Physik Instrumente). The probe had a velocity of 0.7 $\mu m.ms^{-1}$ during the ramp segment of the command for forward motion, and the stimulus was applied for 150 ms. Inward MS currents were recorded at a holding potential of -80 mV. MS currents were characterized by applying series of 0.5 µm or 1 µm incremental steps every 10 s. This was done up to patch rupture.

Pressure-clamp experiments

Patch-clamp experiments were performed under cell-attached configuration (2). Patch pipettes had resistances of 2-3 MΩ when filled with a solution consisting of (in mM) 130 NaCl, 5 KCl, 10 HEPES, 1 $CaCl_2$, 1 $MgCl_2$, 10 TEA-Cl (pH 7.3 with NaOH). External solution used to zero the membrane potential consisted of (in mM) 145 KCl, 10 HEPES, 1 $MgCl_2$, 10 glucose (pH 7.3 with KOH). Currents were sampled at 20 KHz and filtered at 2 KHz. Membrane patches were stimulated with negative pressure pulses through the recording electrode using a Clampex controlled pressure clamp HSPC-1 device (ALA-scientific). Stretch-activated channels were recorded at a holding potential of -80 mV.

Mechanosensitive current analysis

Clampfit 10.7 (Molecular Devices) software was used to analyze recordings and biophysical parameters (2). The decay of inactivation from the peak of current was fitted with mono or bi-exponential function of the form $f(t) = \sum_{i=0}^{n} A_i e^{-t/\tau_i} + C$. Inactivation time constant τ was used to identify the type of MS current components. When fitted with bi-exponential equation, the contribution (in percentage) of each component to the whole current is determined from the ratio of A_i to the amplitude of the peak current.

Animals

Adult male and female C57BL/6 mice (aged 8-12 weeks, sourced from Zhejiang Laboratory Animal Center) were utilized in behavioural investigations. The animals were maintained in

group housing under controlled environmental conditions, with a standardized 12:12 hour light-dark cycle and *ad libitum* access to food and water. Experimental groups were established through randomization procedures and behavioural assays performed blind. The sample size determination was informed by our previous experimental expertise in comparable studies and was deemed statistically adequate. All experimental procedures involving animals were conducted in strict accordance with the guidelines established by the International Association for the Study of Pain and received approval from the Animal Care and Use Committee of Zhejiang Provincial People's Hospital (Protocol No. IACUC-20241119001). All UK experiments were performed in accordance with the UK Animals (Scientific Procedures) Act 1986 with prior approval under a Home Office project licence (PPL 70/7382).

Spared Nerve Injury (SNI) neuropathic pain model

Mice were anesthetized via 1.5% isoflurane inhalation, followed by surgical site preparation including hair removal and sterilization. A longitudinal skin incision was made on the left thigh, and the underlying muscles were carefully dissected to expose the sciatic nerve and its three terminal branches: the common peroneal, tibial, and sural nerves. Following meticulous isolation of these branches, the tibial and common peroneal nerves were ligated and transected, while the sural nerve was preserved intact. The muscle and skin layers were then closed with appropriate sutures. For the sham surgery control group, an identical procedure was performed to expose the sciatic nerve and its branches, but without ligation or transection.

Partial sciatic nerve ligation (PSL) neuropathic pain model

Under isoflurane anaesthesia, partial nerve injury was induced in wild type C57BL/6J adult males by tying a tight ligature with 6-0 silk suture around approximately 1/3 to 1/2 the diameter of the sciatic nerve, similar to the approach described in rats (3). Ipsilateral and contralateral L3-L5 DRGs were dissected prior to RNA isolation on post- surgery day 16.

Sciatic nerve transection (SNT) neuropathic pain model

Under isoflurane anaesthesia, the left sciatic nerve was exposed at thigh level and tightly ligated with 8-0 Prolene non-absorbable suture; 2-4mm of nerve was sectioned distally to prevent the re-joining of the nerve (4). Ipsilateral and contralateral L3-L5 DRGs were dissected three days after surgery.

Behavioural tests

Cotton swab

Prior to behavioural testing, mice were habituated to the von Frey testing apparatus for a period of 60 minutes to minimize stress-induced behavioural alterations. A cotton swab was manually

extended to approximately triple its original length to create an optimal fluffy texture for mechanical stimulation. The mechanical sensitivity was assessed by gently sweeping the cotton swab across the plantar surface of the hindpaw. A positive response was defined as an immediate paw withdrawal upon stimulation. For each mouse, a total of 10 trials were performed, with the response frequency (expressed as the percentage of positive responses out of 10 trials) serving as a quantitative measure of mechanical sensitivity to cotton swab stimulation.

Brush

For the brush test, mice were habituated for one hour in the von Frey test apparatus prior to assessment. The lateral plantar surface of the hindpaw was stimulated by gentle brushing in a heel-to-toe direction using a paintbrush. Behavioural responses were evaluated using a validated scoring system: 0 indicating no response; 1 representing a brief, rapid paw withdrawal; 2 denoting a sustained paw withdrawal exceeding 2 seconds; and 3 signifying more complex behaviours including flinching, licking, or shaking of the stimulated paw.

Von Frey

Mice were allowed to acclimate in the testing chamber for 1 hour before testing. A series of von Frey filaments (ranging from 0.008g to 2g) were applied to the plantar surface of the animal's hind paw using the up-down method and the mechanical threshold for von Frey stimulation was calculated (5).

Pinprick

Prior to behavioural testing, mice were habituated to the von Frey testing apparatus for a period of 60 minutes. The mechanical nociceptive threshold was assessed using a calibrated 27-gauge needle, which was carefully applied to the plantar surface of the hindpaw without breaking the skin integrity. Each experimental subject received a series of 10 discrete mechanical stimuli, administered at one-minute intervals. Positive nociceptive responses were defined as rapid withdrawal of the paw, paw shaking, or paw licking behaviours. The overall response rate was quantified as the percentage of positive responses across the 10 trials, providing a standardized measure of mechanical nociceptive sensitivity.

Randall Selitto

The Randall-Selitto test was conducted by gently restraining each mouse in the experimenter's hand, allowing a 5-minute acclimation period to minimize stress-induced behavioural variations. Subsequently, increasing mechanical pressure was applied to the plantar surface of the hind paw using a calibrated Randall-Selitto apparatus. The maximum force threshold

(cut-off value) was established at 300 grams to prevent tissue damage. A positive behavioural response was defined as either a visible withdrawal of the hind limb or an audible vocalization. The minimum force (in grams) required to elicit such a response was recorded as the mechanical nociceptive threshold.

Hot plate

The hot plate test was conducted by placing the mouse on a temperature-controlled hot plate maintained at a constant temperature of 50°C. The latency to exhibit nociceptive behaviours, including hind paw lifting, licking, shaking, or spontaneous jumping, was carefully monitored. Upon observation of any of these pain-related responses, the mouse was immediately removed from the hot plate surface to prevent tissue damage. The elapsed time from placement to the first manifestation of nociceptive behaviour was recorded as the pain response latency.

Hargreaves'

For the Hargreaves' test, experimental mice were individually placed in transparent plexiglass chambers positioned on an elevated glass platform and allowed to acclimate to the testing environment for 60 minutes. A calibrated infrared heat source was positioned beneath the glass surface, directly targeting the plantar surface of the animal's hind paw. The latency to nociceptive behaviours (characterized by paw withdrawal, licking, or shaking) was recorded. To prevent potential tissue injury, a 30-second cut-off limit was automatically implemented. Each subject underwent three independent trials, separated by 5-minute intervals to avoid sensitization. The mean latency of these three measurements was calculated and used for subsequent analysis.

Acetone

Prior to testing, mice were placed individually in transparent observation chambers (von Frey test box) and allowed to acclimate to the testing environment for a period of one hour. For the assessment of cold sensitivity, a 1 ml syringe was utilized to deliver a single drop of acetone to the plantar surface of the hind paw. The behavioural responses were quantified using a standardized scoring system: 0 indicating no observable response; 1 representing a mild response characterized by paw withdrawal or a single shake; 2 denoting moderate responsiveness manifested as repeated paw shaking; and 3 indicating the most pronounced response involving paw licking behaviour.

Cold plantar assay

The animals were placed in a transparent test chamber equipped with a glass floor and allowed to acclimate for a period of 60 minutes prior to experimental procedures. A modified 5ml syringe, with its nozzle portion removed, was filled with dry ice particles. Consistent pressure was applied to the syringe while positioning the dry ice tip against the glass floor, directly beneath the centre of the animal's hindpaw. The withdrawal latency, defined as the time elapsed until the hindpaw was withdrawn from the cold stimulus was recorded. Three independent measurements were taken for each animal, with a 10-minute recovery interval between successive trials. The final withdrawal latency was calculated as the arithmetic mean of these three measurements.

Rotarod

The rotarod apparatus was configured with an initial rotational velocity of 5 revolutions per minute (rpm), programmed to accelerate progressively to a terminal velocity of 40 rpm over a 180-second interval. The experimental protocol involved measuring the duration (latency) until the subject's involuntary dismount from the rotating cylinder. To ensure statistical reliability and account for potential variability in performance, each experimental subject was evaluated through a series of three consecutive trials, with standardized 10-minute rest periods implemented between successive trials. The final performance metric was derived from the arithmetic mean of these three independent measurements.

Spontaneous pain assessment

Spontaneous pain behaviour was evaluated 4 weeks after intrathecal injection of *MDFIC2*-AAV, which was administered 1 week after SNI surgery to allow sufficient time for viral expression. The assessment was performed as described in previous study (6). Briefly, mice were placed individually in transparent Plexiglass chambers and videotaped for 30 minutes over three consecutive mornings to allow for habituation. Spontaneous pain behaviour was quantified using the recording from the third day. On the same afternoon, animals received an intraperitoneal injection of phenylephrine (0.6 mg/kg) or vehicle, and behaviour was recorded for 60 minutes. Pain-related behaviours, specifically licking (2 points per bout) and flinching/shaking/lifting (1 point per bout) of the ipsilateral hind paw-were scored from minute 15 to minute 45 post-injection to avoid injection-related stress artifacts. Behavioural scoring was performed by an experimenter blinded to treatment groups. Spontaneous pain scores were calculated as the total points accumulated during the 30-minute observation period.

Viral vectors

The recombinant adeno-associated virus serotype 9 (AAV9) vectors were obtained from VectorBuilder. Two sets of viral constructs were utilized: (1) AAV9 CAG-h*MDFIC2*-IRES-

mCherry and its corresponding control AAV9 CAG-mCherry plasmids, and (2) AAV9 CAG-mCherry-U6-m*Mdfic2*_shRNA and its control AAV9-CAG-mCherry-U6-Scramble_shRNA plasmids. The m*Mdfic*2 shRNA targeting sequence was designed as GCAGACGAGAAACCTATTAAT, while the non-targeting Scramble shRNA sequence was CCTAAGGTTAAGTCGCCCTCG. The AAV vectors were produced by standard triple transfection of HEK293 cells and purified by ultracentrifugation of density gradients. Following viral packaging and purification, the viral titers were quantified and determined to be in excess of 1.0×10^{13} genomic copies per milliliter (GC/ml) for both viral vectors.

Intrathecal injection of viral vectors

Mice were anesthetized via inhalation of 1.5% isoflurane and positioned prone on a thermoregulated heating pad with elevated pelvic region. Using a Hamilton microsyringe (10 µL capacity), the designated viral vector solution was carefully aspirated. The needle was then precisely inserted into the subarachnoid space between the L4 and L5 vertebrae. Successful penetration into the subarachnoid space was confirmed by the presence of a characteristic tail-flick reflex. Subsequently, the viral vector solution was administered via slow injection, and the needle was maintained in position for an additional 30 seconds post-injection before withdrawal. Following behavioural assessments, the expression of viral vectors in dorsal root ganglia tissue was validated using qRT-PCR.

qRT-PCR

After deep anaesthesia administration via isoflurane inhalation, mice were humanely euthanized through cervical dislocation. Subsequently, L4-L5 dorsal root ganglia or L3-L5 DRG (SNT and PSL models) were carefully isolated. Total RNA extraction was performed using commercial RNA extraction reagents following the manufacturer's protocol. The isolated RNA was reverse transcribed to complementary DNA (cDNA) using a reverse transcription kit and thermal cycler under standard conditions. The mRNA expression levels of target genes were quantitatively assessed using real-time RT-PCR. The qPCR reaction mixture (20 µl final volume) was prepared containing equivalent amounts of reverse transcription products, 10 µl of premix, and gene-specific primers at a final concentration of 200 nM each. Target mRNA levels were analyzed using threshold cycle (Ct) values and normalized to β-actin as an internal reference gene. Relative gene expression was calculated using the comparative $2^{-\Delta\Delta Ct}$ method. The following primer sequences were utilized for PCR amplification: Human *MDFIC2* (h*MDFIC2*), Forward: 5'-GACTTCAATATCACAGATGGACCAG-3', Reverse: 5'-ATTTTGGTACACACCGTTTCGCAG-3'; Mouse *Mdfic2* (m*Mdfic*2), Forward: 5'-TGCCAAACGAAATTTCCCTACCTC-3', Reverse: 5'-TGATGCACACTGTCTCACACGTG-3';

Mouse β-actin (mβ-actin), Forward: 5'-GACGTTGACATCCGTAAAGA-3', Reverse: 5'-AATCTCCTTCTGCATCCTGT-3'.

Model Fitting and 3D illustrations

3D structural model of Mdfic2 was obtained from AlphaFold (https://alphafold.ebi.ac.uk/). Three parts of the AlphaFold models (aa 79-189, AF-B2RVL9-F1-v4) were fitted into EM 3D structure of Piezo1 complexed with three Mdfic C-terminal helices (PDB ID: 8imz and EMD-35577 map) (7). Illustrations and movie were generated using PyMOL (version 3.04 https://www.pymol.org/).

Statistical analyses

Electrophysiology. Except for current kinetics, which were fitted with Clampfit 10 (Axon Instruments), and full-point histograms used for unitary current characterization, which were fitted with Igor Pro 6 (WaveMetrics), data were analyzed using GraphPad Prism 8.2.0 (San Diego, USA). Statistical details can be found in the figure legends and in the main text. Reported n values can be found in the figure legends or in the results. Data are represented as box and whiskers (min to max) or as mean ± SEM. (standard error of mean). All replicates were biological. Sample sizes were not *a priori* determined and are in line with standards in the field. All statistical tests are indicated in the respective figure legend and are two-sided. In all figure legends the exact value and definition of n is indicated. In all panels: not significant, ns $p > 0.05$; * $p < 0.05$; ** $p < 0.01$; *** $p < 0.001$; **** $p < 0.0001$.

Behaviour experiments. All statistical analyses were conducted using GraphPad Prism software. Data are expressed as mean ± standard deviation (SD). The normality of data distribution was assessed using the Shapiro-Wilk test, while homogeneity of variance was evaluated using Levene's test. For two-group comparisons, statistical significance was determined using either Student's t-test for parametric data or Mann-Whitney U test for non-parametric data. Multiple group comparisons were performed using two-way analysis of variance (ANOVA) followed by Bonferroni's post hoc test for parametric data, or Kruskal-Wallis test followed by Dunn's multiple comparisons test for non-parametric data. A probability value of $p < 0.05$ was considered statistically significant for all analyses.

References

1. A. E. Dubin *et al.*, Endogenous Piezo1 Can Confound Mechanically Activated Channel Identification and Characterization. *Neuron* **94**, 266-270 e263 (2017).
2. T. Parpaite *et al.*, Patch-seq of mouse DRG neurons reveals candidate genes for specific mechanosensory functions. *Cell Rep* **37**, 109914 (2021).

3. Z. Seltzer, R. Dubner, Y. Shir, A novel behavioral model of neuropathic pain disorders produced in rats by partial sciatic nerve injury. *Pain* **43**, 205-218 (1990).
4. P. D. Wall *et al.*, Autotomy following peripheral nerve lesions: experimental anaesthesia dolorosa. *Pain* **7**, 103-113 (1979).
5. S. R. Chaplan, F. W. Bach, J. W. Pogrel, J. M. Chung, T. L. Yaksh, Quantitative assessment of tactile allodynia in the rat paw. *J Neurosci Methods* **53**, 55-63 (1994).
6. W. Xie *et al.*, Vascular motion in the dorsal root ganglion sensed by Piezo2 in sensory neurons triggers episodic neuropathic pain. *Neuron* 10.1016/j.neuron.2025.03.006 (2025).
7. Z. Zhou *et al.*, MyoD-family inhibitor proteins act as auxiliary subunits of Piezo channels. *Science* **381**, 799-804 (2023).

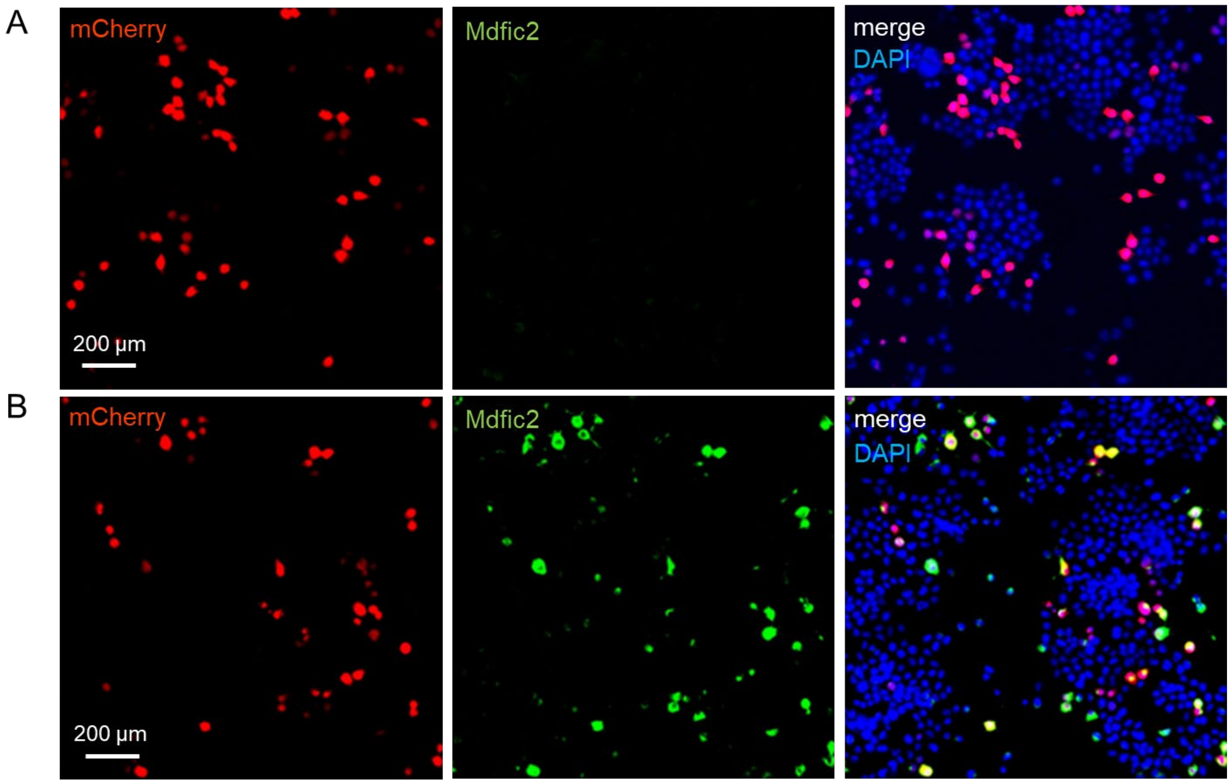

Figure S1. Rabbit polyclonal anti-Mdfic2 immunostaining on Cad cells. **A.** Cells transfected with mCherry plasmid. Cad cells do not endogenously express Mdfic2 and no anti-Mdfic2 staining is detected. **B.** Cells transfected with HA-MDFIC2-IRES-mCherry plasmid. Transfected cells (indicated by mCherry red fluorescence) detected with anti-Mdfic2 antibody (green). DAPI staining in blue.

```
Human       MSETELEKIKVRTAEHLENDKNNISWLKEDTQLTNAKHADEKPINAIVINSVSDFNITDG 60
Mouse       MSEIELEKIKVRTAEHFENGKNNLPWLKEDTQLTNEKHADEKPINAIVINSVSEFSITDG 60
            *** ************:**.***: ********** *****************:*.****

Human       PAKENPNEKKLSESSTSLSSLEECQTTFSYLQTDTSVHHRDTDEECASLILACLFCQFWD 120
Mouse       PAQETPNEKNLSGSSTSLSSLEECQTKFPYLQTNTSVHRRDADEECASLVLTCLFCQFLD 120
            **:*.****:** *************.* ****:****:**:*******:*:****** *

Human       CLLMLPGTCETVCTKMCCPSHRYHHTSDENHSRNDCSCNCDMDCSLFESCHETSECLELA 180
Mouse       CLFMLPDTCETVCINLCCPSHRYYSASDENHPHNDCNLTCDVDCSLFESCHETSECLELA 180
            **:***.****** ::*******: :***** :***. .**:******************

Human       MEISEICYR    189
Mouse       MEISEICYR    189
            *********
```

Figure S2. Amino acid sequence alignment for human MDFIC2 (AHA59118) and mouse Mdfic2 (NP_001121564) showing 82% identity and 87% similarity. Amino acid residues are coloured to help the visualisation of their chemistry. Negatively charged amino acid residues are shown in blue, hydrophobic amino acid residues in red, positively charged amino acid residues in magenta, and polar amino acid residues are shown in green.

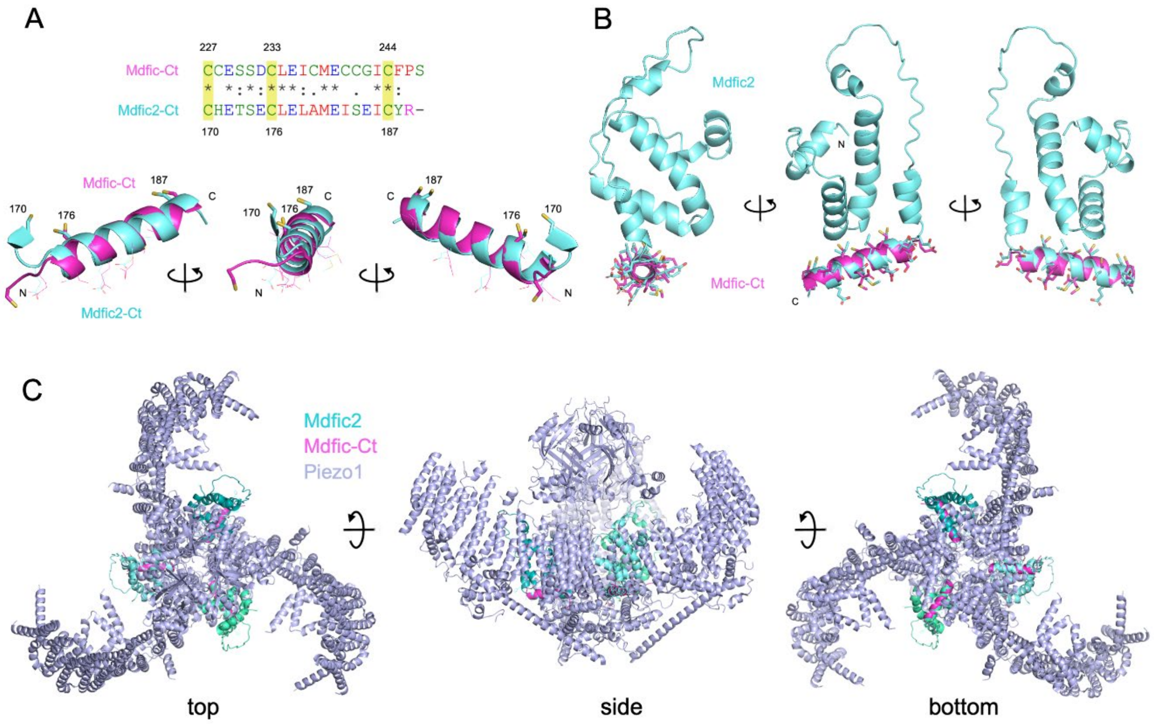

Figure S3. AlphaFold modelling shows Mdfi/Mdfic interaction with Piezo1 is conserved in Mdfic2

(**A**) Amino acid sequences of C-terminal alpha helices of Mdfic and Mdfic2 proteins show high degree of conservation. Three cysteine amino acid residues in Mdfic2-Ct α-helix (in cyan; C170, C176 and C187 - mouse Mdfic2 numbering corresponding to C227, C233 and C244 in mouse Mdfic) are conserved when compared to seven cysteines in the Mdfic-Ct (magenta) and face the same orientation in the C-terminal α-helix. Conserved cysteine residues are shown as sticks and conserved negatively charged amino acids (Asp and Glu) are shown as lines. N and C indicate N- and C- termini of the α-helices. Numbering is shown for Mdfic2 cysteines. **(B)** AlphaFold model of Mdfic2 (in cyan) shows helical structure of the C-terminal part of the protein (Ser79 to Arg189) made of seven α-helices (110 C-terminal amino acids) connected by loops. The first 23 amino acids form a long α-helix connected to the rest of the protein by an unstructured loop made of 55 amino acids (see PDB file provided in Dataset). The Mdfic C-terminal α-helix aligned with the model is shown in magenta. N and C indicate N- and C- termini of the protein. Conserved amino acid residues are shown as sticks. **(C)** Three molecules of Mdfic2 (Ser79-Arg189) in cyan hues, AlphaFold models) are fitted into EM 3D structure of Piezo1 (in slate blue, PDB: 8imz) complexed with three C-terminal α-helices of Mdfic (in magenta). All three molecules of Mdfic2 are aligned with the respective C-terminal α-helices of Mdfic. The alignment shows sufficient availability of space for Mdfic2 molecules to be bound to the Piezo1 trimer between the “blades” of the structure without sterical clashes – as also can be viewed in Video S1.

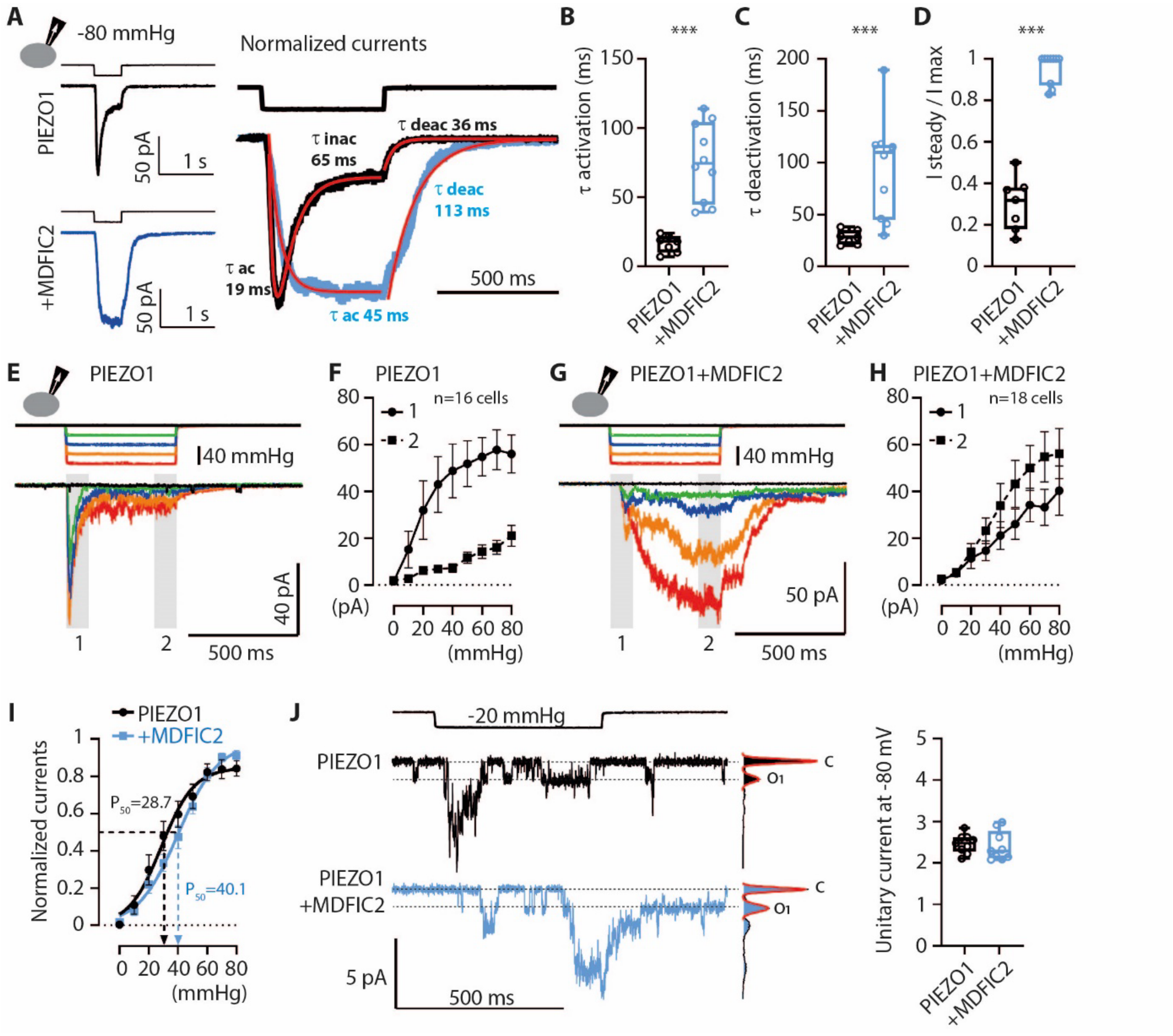

Figure S4. Pressure-clamp characterization of MDFIC2 modulation of PIEZO1 mediated currents.

A. Typical averaged current traces from PIEZO1 ± MDFIC2 transfected cells evoked by -80 mmHg pressure stimulation (left panel). Current kinetics are fitted with exponential equations (right panel, red traces). **B-D.** Time constants of activation (**B**), deactivation (**C**), and the ratio of current remaining at the end of the 500 ms stimulation (**D**). N = 7 and 10 cells for PIEZO1 and PIEZO1 + MDFIC2, respectively. ***: $p < 0.001$, Mann Whitney test. Measures are based on 6-10 averaged traces. **E-H**. Current pressure relationships of PIEZO1 ± MDFIC2 transfected cells. Typical current traces of currents evoked by incremental pressure steps from 0 to -80 mm Hg in PIEZO1 (**E**) and PIEZO1+MDFIC2 (**G**) transfected cells. Gray boxes highlight the first 100 ms (1) or last 100 ms (2) of the 500 ms pressure stimulation used for measures represented in **F** and **H**. Average current pressure relationships at the beginning (1) and at the end (2) of the pressure stimulation, in PIEZO1 (**F,** n = 16 cells) and PIEZO1 + MDFIC2 (**H**, n = 18 cells) transfected cells (mean ± s.e.m.). **I.** Current pressure relationships of PIEZO1 ± MDFIC2 transfected cells fitted with Boltzmann equation. Currents were normalized to Imax for each cell individually prior averaging (mean ± s.e.m.). P_{50} values of fitted curves are statistically different (F-test, $p = 0.0013$). **J**. Examples of recordings from PIEZO1 ± MDFIC2 transfected cells used to determine unitary currents (left panel). "c" corresponds to the closed state and "o1" to the single channel open state. The comparison of unitary currents of PIEZO1 channels ± MDFIC2 is shown in the right panel (n = 9 cells for each condition). All recordings are made at Vh = -80 mV in HEK-P1KO cells.

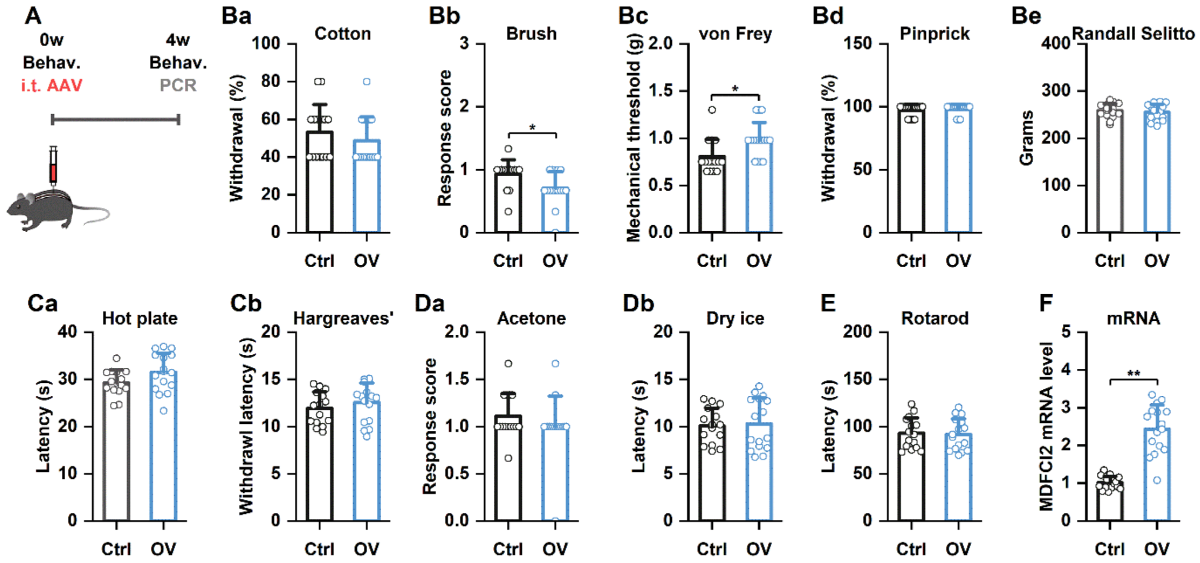

Figure S5. *MDFIC2* overexpression attenuates mechanical hypersensitivity to brush and von Frey filament stimulation in naive mice. A. Schematic representation of the experimental design and timeline. **B.** Quantitative analysis demonstrating the effects of *MDFIC2* overexpression on mechanical sensitivity assessed through cotton swab (**Ba**, unpaired *t* test, $P = 0.35$), brush (**Bb**, unpaired *t* test, *$P = 0.017$), von Frey filaments (**Bc**, unpaired *t* test, *$P = 0.012$), pinprick (**Bd**, unpaired *t* test, $P = 0.61$), and pressure stimulation (**Be**, unpaired *t* test, $P = 0.58$) in mice. **C.** Assessment of thermal nociception following *MDFIC2* overexpression using hot plate (**Ca**, unpaired *t* test, $P = 0.08$) and radiant heat (**Cb**, unpaired *t* test, $P = 0.39$) tests. **D.** Evaluation of cold sensitivity following *MDFIC2* overexpression using acetone evaporation (**Da**, unpaired *t* test, $P = 0.29$) and cold plantar (**Db**, unpaired *t* test, $P = 0.79$) tests. **E.** Assessment of motor function following *MDFIC2* overexpression (unpaired *t* test, $P = 0.83$). **F.** Quantitative real-time PCR analysis confirming successful *MDFIC2* overexpression (unpaired *t* test, **$P < 0.01$). N = 15 and 16 mice for each group. Ctrl: control, OV: overexpression.

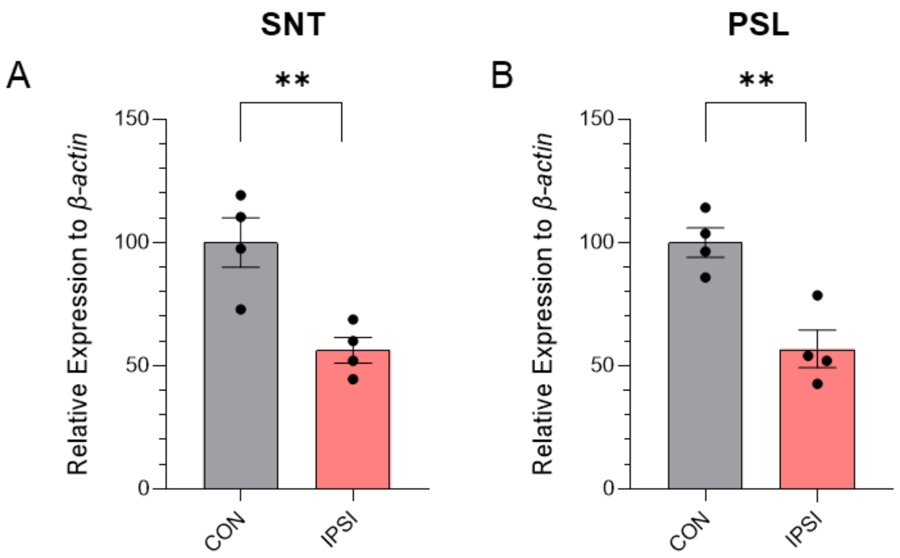

Figure S6. *Mdfic2* is significantly downregulated in dorsal root ganglia in two models of neuropathic pain

Expression level of *Mdfic2* in L3-L5 DRGs following SNT and PSL neuropathic pain models in wild type C57BL/6J adult males. **A.** *Mdfic2* expression in operated (red, ipsilateral) and non-operated (grey, contralateral) side of L3-L5 DRGs 3 days following spinal nerve transection (SNT) (n=4); **B.** *Mdfic2* expression in operated (red, ipsilateral) and non-operated (grey, contralateral) side of L3-L5 DRGs 16 days following partial sciatic nerve ligation (n=4). Data-points are denoted by dots, bars show the ± SEM, and data analysed by a Student's *t*-test ***P*<0.01.

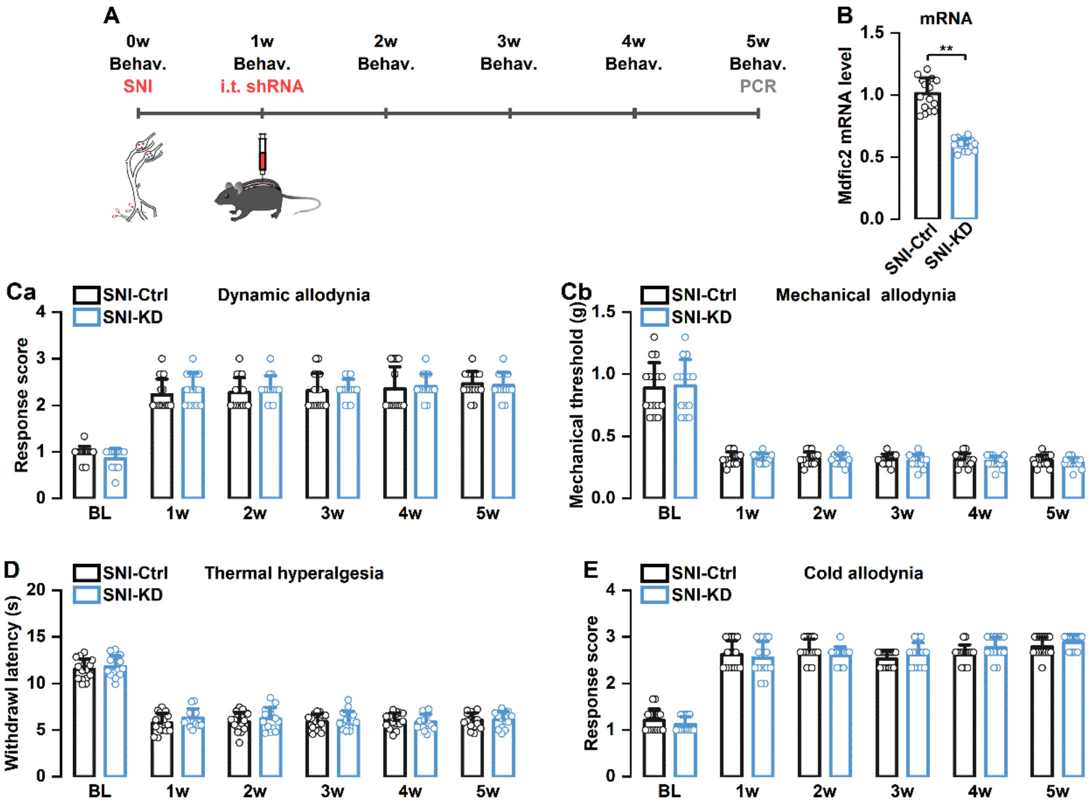

Figure S7. ***Mdfic2*** **reduction demonstrated no effects on established neuropathic pain in SNI model mice. A.** Experimental protocol timeline illustrating shRNA administration, SNI operation and behavioural assessment sequence. **B.** RT-qPCR validation demonstrating successful *Mdfic2* suppression (unpaired *t* test, $^{**}P < 0.01$). **C.** Administration of *Mdfic2* shRNA showed no significant impact on dynamic (**Ca**) and static (**Cb**) mechanical sensitivity following SNI. Two-way repeated-measures ANOVA, Bonferroni post hoc test, adjusted $P > 0.05$. **D.** *Mdfic2* suppression via shRNA demonstrated no observable changes in heat hypersensitivity post-SNI. Two-way repeated-measures ANOVA, Bonferroni post hoc test, adjusted $P > 0.05$. **E.** Cold sensitivity following SNI remained unaltered by *Mdfic2* shRNA treatment. Two-way repeated-measures ANOVA, Bonferroni post hoc test, adjusted $P > 0.05$. N=16 and 14 mice for each group. Ctrl: control, KD: knockdown, BL: baseline.

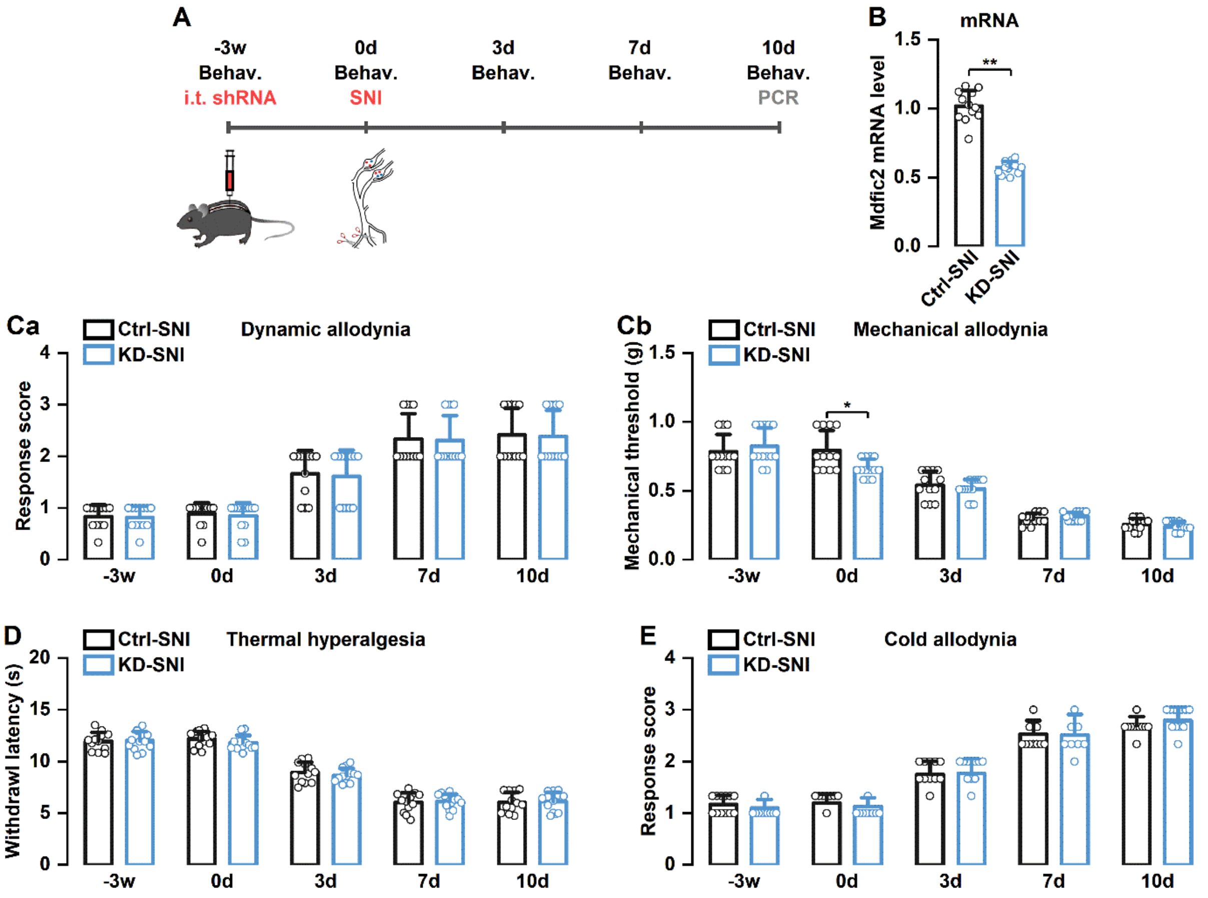

Figure S8. ***Mdfic2* reduction did not prevent neuropathic pain progression in SNI model mice. A.** Experimental protocol timeline illustrating shRNA administration, SNI operation and behavioural assessment sequence. **B.** RT-qPCR validation demonstrating successful *Mdfic2* suppression (unpaired *t* test, $^{**}P < 0.01$). **C.** Administration of *Mdfic2* shRNA showed no significant impact on dynamic (**Ca**) and static (**Cb**) mechanical sensitivity following SNI. Two-way repeated-measures ANOVA, Bonferroni post hoc test, adjusted $^{*}P = 0.013$. **D.** *Mdfic2* suppression via shRNA demonstrated no observable changes in heat hypersensitivity post-SNI. Two-way repeated-measures ANOVA, Bonferroni post hoc test, adjusted $P > 0.05$. **E.** Cold sensitivity following SNI remained unaltered by *Mdfic2* shRNA treatment. Two-way repeated-measures ANOVA, Bonferroni post hoc test, adjusted $P > 0.05$. N=12 and 13 mice for each group. Ctrl: control, KD: knockdown.

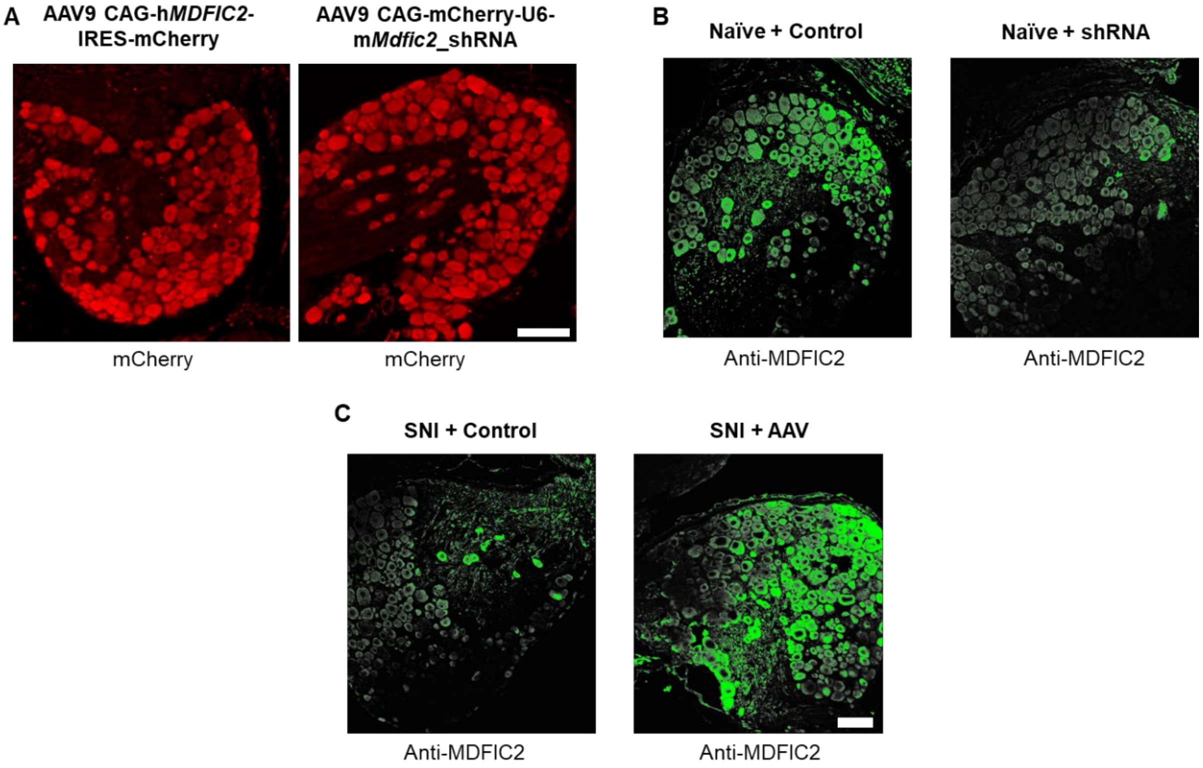

Figure S9. AAV delivery in DRG. A. Representative images showing the expression of mCherry (red) in DRG of AAV9 CAG-h*MDFIC2*-IRES-mCherry or AAV9 CAG-mCherry-U6-m*Mdfic2*_shRNA treated mice at 4 weeks post-intrathecal injection. **B.** Anti-MDFIC2 staining (green) in DRG tissues from naïve mice collected 4 weeks after intrathecal injection of AAV9-CAG-mCherry-U6-Scramble_shRNA (control) or AAV9 CAG-mCherry-U6-m*Mdfic2*_shRNA. Note the reduced MDFIC2 protein after shRNA treatment. **C.** Anti-MDFIC2 staining in DRG tissues from mice that underwent SNI surgery followed by intrathecal injection of AAV9 CAG-mCherry (control) or AAV9 CAG-h*MDFIC2*-IRES-mCherry at 1 week post-surgery. DRG tissues were harvested 4 weeks after viral injection and immunofluorescence staining was performed. Scale bar = 200 μm.

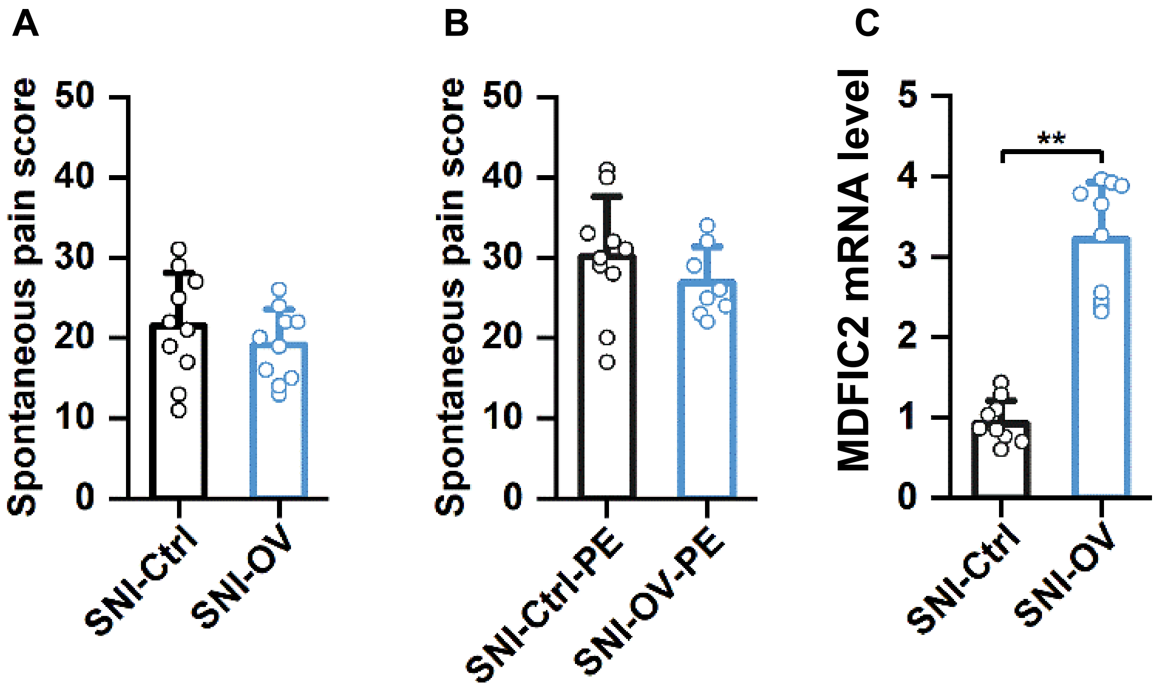

Figure S10. Effect of *MDFIC2* overexpression on spontaneous pain in SNI mice. **A.** AAV-mediated overexpression of *MDFIC2* (OV) did not reduce spontaneous pain behaviour following spared nerve injury (SNI) in mice. *N* = 10 per group. **B.** Following intraperitoneal injection of phenylephrine (PE, 0.6 mg/kg) to induce spontaneous pain behaviour, *MDFIC2* overexpression still did not significantly affect spontaneous pain in SNI mice. *N* = 10 per group. **C.** RT-qPCR analysis confirmed successful upregulation of *MDFIC2* expression in DRG tissues following AAV-mediated overexpression (unpaired *t* test, $^{**}P < 0.01$).

Video S1: AlphaFold modelling of Piezo1 and Mdfic2

Three molecules of Mdfic2 ((Ser79-Arg189) in cyan hues, AlphaFold models) are fitted into EM 3D structure of Piezo1 (in slate blue, PDB: 8imz) complexed with three C-terminal α-helices of Mdfic (in magenta). All three molecules of Mdfic2 are aligned with the respective C-terminal α-helices of Mdfic.

Dataset: PDB coordinates file of the Mdfic2 (Gm765) AlphaFold model
